# Electroacupuncture enhances anti-tumor immunity in TNBC by reducing NE release and blocking α2-AR-mediated NGF/Hippo signaling

**DOI:** 10.1186/s13020-026-01445-6

**Published:** 2026-07-13

**Authors:** Fei-Fei Li, Yan Huang, Chun-Fang Gao, Chun-Yu Wu, Chen-Ping Sun, Yue-Nong Qin, Ying Xie, Sheng Liu, Huan-Gan Wu

**Affiliations:** 1 Yueyang Hospital of Integrated Traditional Chinese and Western Medicine, Shanghai University of Traditional Chinese Medicine, Shanghai, 200437 People’s Republic of China; 2 Integrated Traditional Chinese and Western Medicine Breast Department, Longhua Hospital Shanghai University of Traditional Chinese Medicine, Shanghai, 200030 People’s Republic of China; 3 Shanghai Institute of Acupuncture and Meridian Research, Shanghai, 200030 People’s Republic of China; 4 Institute of Surgery, Longhua Hospital Shanghai University of Traditional Chinese Medicine, Shanghai, 200030 People’s Republic of China

**Keywords:** Electroacupuncture, Triple-negative breast cancer, Norepinephrine, Tumor immune microenvironment, CD8^+^ T cell

## Abstract

**Background:**

Targeting the neuro-immune microenvironment to suppress triple-negative breast cancer (TNBC) represents a critical strategy in tumor immunotherapy.

**Methods:**

A syngeneic 4T1 orthotopic TNBC model in Balb/c mice was employed. EA was applied at ST36 (Zusanli) using systematically optimized parameters (2/15 Hz, 3 mA, 30 min, every other day). Multi-dimensional immunophenotyping by flow cytometry, immunofluorescence, and Western blot was performed across tumor, blood, and splenic compartments. Transcriptome sequencing coupled with KEGG/GSEA pathway analysis was used to identify downstream signaling networks. The NGF/Hippo/YAP axis and adrenergic receptor subtype specificity were validated through pharmacological intervention in vitro, while EA synergy with αPD-L1 was assessed in a CD8^+^ T cell-depletion model.

**Results:**

ST36 stimulation at 3 mA preferentially suppressed TNBC tumor growth and augmented intratumoral immune infiltration, characterized by elevated CD8^+^ T cells, NK cells, and M1-polarized macrophages. EA significantly enhanced CD8^+^ T cell effector capacity, upregulating perforin, granzyme B, CD69, and ZAP70 phosphorylation, and synergized with αPD-L1 in a CD8^+^ T cell-dependent manner. Mechanistically, EA activated c-Fos^+^/ChAT^+^ cholinergic neurons in the dorsal motor nucleus of the vagus (DMV) and reduced norepinephrine (NE) output in both circulation and tumors. Transcriptomic profiling identified NGF downregulation and Hippo pathway activation as central events. EA upregulated AMOT, driving YAP phosphorylation at Ser127, cytoplasmic sequestration of YAP, and suppression of downstream IL-6 secretion. In vitro, exogenous NGF suppressed YAP phosphorylation and promoted TNBC malignant behavior, effects fully reversed by the YAP inhibitor verteporfin. α2-adrenergic receptor (α2-AR) antagonism with yohimbine abrogated NE-induced NGF upregulation, pinpointing α2-AR as the receptor subtype linking sympathetic signaling to the NGF/Hippo axis.

**Conclusion:**

EA at ST36 recalibrates neuro-sympathetic tone in TNBC by activating vagal cholinergic outflow, reducing NE-driven α2-AR/NGF/Hippo signaling, and thereby relieving immunosuppression while amplifying CD8^+^ T cell-mediated cytotoxicity. These findings establish a neuro-immune mechanistic framework for EA-based adjuvant immunotherapy in TNBC.

**Supplementary Information:**

The online version contains supplementary material available at 10.1186/s13020-026-01445-6.

## Introduction

Breast cancer remains one of the leading causes of cancer-related mortality among women globally [1]. Triple-negative breast cancer (TNBC), an aggressive and biologically heterogeneous subtype, is defined by the absence of estrogen receptor (ER), progesterone receptor (PR), and human epidermal growth factor receptor 2 (HER2), accounting for approximately 15–20% of all breast cancer cases [2, 3]. Although immune checkpoint inhibitors (ICIs) have shown promise in treating TNBC [4], there are significant individual differences in patients’ responses to immunotherapy. Consequently, TNBC treatment continues to face substantial challenges and is widely recognized as a clinically refractory malignancy [5]. This underscores the urgent need for more effective combination therapies in TNBC.

The regulation of the immune microenvironment is intimately linked to TNBC prognosis [6, 7]. Evidence indicates that breast cancer patients achieving pathological complete response (pCR) after neoadjuvant chemotherapy exhibit robust T cell infiltration in tumor tissues [8]. A large cohort study involving thousands of patients further revealed that dense infiltration of CD8^+^ T cells and tumor-infiltrating lymphocytes (TILs) significantly correlates with prolonged 10-year disease-free survival (DFS) and overall survival (OS) in breast cancer patients [9]. These findings collectively suggest that targeted modulation of the immune microenvironment represents a critical strategy for enhancing TNBC clinical efficacy.

Acupuncture, a cornerstone of Traditional Chinese Medicine (TCM), is known for its ability to regulate systemic immunity [10]. Through principles such as balancing yin and yang, invigorating qi and blood, and strengthening visceral functions, it exerts a crucial regulatory effect on the body’s defense mechanisms. In recent years, research has revealed that acupuncture can also enhance immune function by modulating the neuro-endocrine-immune (NEI) network, thereby eliciting anti-tumor effects. For instance, electroacupuncture (EA) in breast cancer mouse models activates vagal nerve conduction, consequently enhancing the proportion and function of CD8^+^ T cells and NK cells [11]. In colorectal cancer, EA activates anti-tumor immunity in tumor-bearing mice, significantly increasing CD8+ T cell infiltration, promoting lymphocyte and granzyme B expression in tumor tissues, and augmenting the therapeutic efficacy of programmed cell death-1 (PD-1) inhibitors [12].

However, most contemporary EA research primarily addresses chronic, inflammatory, and pain-related conditions. While its application in oncology largely concentrates on alleviating treatment-related side effects and improving patient quality of life, the direct anti-tumor effects and underlying mechanisms of EA remain less explored. To bridge this gap, our study aimed to investigate the direct inhibitory effect of EA on TNBC through in vitro and in vivo experiments. We sought to identify effective acupoints and intervention parameters, explore the mechanistic basis of EA’s action, and elucidate the scientific underpinnings of its efficacy. This research ultimately provides robust scientific evidence for EA’s role in augmenting anti-tumor immunity, offering a novel perspective for TNBC immunotherapy.

## Materials and methods

### Animals

Specific pathogen-free (SPF) healthy female Balb/c mice, aged 6–8 weeks with a body weight of (19 ± 1) g, were purchased from Shanghai Slack Laboratory Animal Co., Ltd. The mice were housed in a specific-pathogen-free animal laboratory with free access to food and water. The housing conditions were maintained at a temperature of 18–22 °C and a 12 h light/12 h dark cycle. The study protocol was approved by the Animal Ethics Committee of Longhua Hospital Affiliated to Shanghai University of Traditional Chinese Medicine (Ethics No.: LHERAW-25053).

### Cell lines

The mouse TNBC cell line 4T1 was cultured in RPMI-1640 medium (Gibco, Carlsbad, CA, USA), the human TNBC cell line MDA-MB-231 was cultured in Dulbecco's Modified Eagle Medium (DMEM) (Gibco, Carlsbad, CA, USA). All cell culture media were supplemented with 10% Fetal Bovine Serum (FBS) and 1% penicillin–streptomycin and were maintained at 37 °C in a humidified atmosphere of 5% CO_2_. The 4T1-luc cell line was generated by transducing 4T1 cell with lentiviruses carrying a luciferase gene, followed by selection with antibiotics to obtain a stable luciferase-expressing clone. All cell lines were purchased from the Chinese Academy of Sciences Cell Bank and tested negative for mycoplasma contamination.

### TNBC mouse model

4T1 was chosen as the TNBC model for its well-characterized syngeneicity, invasiveness, and mirroring human TNBC’s aggressiveness. Critically, it is compatible with immunocompetent Balb/c mice, preserving the intact host immune microenvironment. 4T1-luc cells in the logarithmic growth phase were digested with trypsin, centrifuged at 1000 rpm for 5 min, and the supernatant was discarded. The cells were then resuspended in phosphate-buffered saline (PBS). Each mouse was subcutaneously injected with 3 × 10^4^ 4T1-luc cells into the left mammary fat pad. Tumor volume was measured regularly using the formula: V = 0.5 × a × b^2^, where V represents tumor volume, a denotes the maximum tumor diameter, and b denotes the minimum tumor diameter. The model was considered successfully established when the tumor volume reached over 100 mm^3^ approximately 2 weeks after injection.

### CD8^+^ T cell depletion TNBC model

After successful establishment of the TNBC mouse model, mice in the CD8^+^ T cell depletion group were intraperitoneally injected with anti-mouse CD8α antibody (BE0061, BioXCell, USA) at a dose of 200 μg per injection, once a week for 3 consecutive weeks. This intervention achieved targeted depletion of CD8^+^ T cells in the tumor microenvironment, providing a critical model basis for evaluating the role of CD8^+^ T cells in anti-tumor immunity.

### EA intervention

EA was performed using a HANS-200A Korean-style electroacupuncture instrument (Nanjing Jisheng Medical Co., Ltd., China) with the following parameters: 2/15 Hz, a current intensity of 1 mA or 3 mA, and the intervention was administered once every other day. Murine ST36 is located on the posterolateral aspect of the knee joint, approximately 2 mm inferior to the head of the fibula; murine ST25 is located on the abdomen, 3 mm lateral to the midline.

### Experimental drugs

InVivoMab anti-mouse programmed death-ligand 1 (PD-L1) (cat. no. BE0101, BioXCell, USA). β-nerve growth factor (NGF) (cat. no. 450-01, Peprotech, US): A stock solution of human β-NGF was prepared in sterile water at a concentration of 100 μg/ml, and then a working solution at 10 μg/ml was stored in phosphate-buffered saline (PBS) containing 5% trehalose. Verteporfin (VP) (cat. no. HY-B0146, MedChemExpress, China); norepinephrine hydrochloride (cat. no. HY-13715A, MedChemExpress, China); β2-adrenergic receptor (AR) antagonist (ICl-118551 hydrochloride) (cat. no. HY-13951, MedChemExpress, China); α2-AR antagonist (yohimbine hydrochloride) (cat. no. HY-NO127, MedChemExpress, China).

### Flow cytometry

Peripheral blood was collected via orbital puncture, treated with red blood cell lysis buffer on ice for 5 min, centrifuged (1500 rpm, 5 min, 4 °C), resuspended in PBS, blocked with 2% BSA at 4 °C for 30 min, and 1 × 10^6^ cells were stained with fluorochrome-conjugated antibodies (30 min, room temperature, dark) before washing and analysis. For tumor tissues, minced samples were digested in pre-prepared buffer at 37 °C for 40 min (shaken every 10 min), centrifuged, filtered through a 40 μm strainer, resuspended in 2% FBS-PBS, counted, and 10⁷ cells were prepared for staining. Spleens were minced, filtered, centrifuged, resuspended in 2% FBS-PBS, counted, and 10^7^ cells were processed similarly. Lymphoid cells were stained with anti-mouse CD8a-APC (cat. no. 100712, BioLegend, USA), anti-mouse CD3-PE (cat. no. 100205, BioLegend, USA), anti-mouse CD4-FITC (cat. no. 100405, BioLegend, USA), anti-mouse CD45-BV421 (cat. no. 147719, BioLegend, USA), and anti-mouse CD49b-PE-Cy7 (cat. no. 108922, BioLegend, USA); myeloid cells were stained with anti-mouse CD45-BV421 (cat. no. 147719, BioLegend, USA), anti-mouse CD11b-PerCP-Cy5.5 (cat. no. 45-0112-82, Invitrogen, USA), anti-mouse MHC II-BV510 (cat. no. 107635, BioLegend, USA), anti-mouse CD86-APC (cat. no. 17-0862-82, Invitrogen, USA), and anti-mouse F4/80-PE/Cy7 (cat. no. 123113, BioLegend, USA). After antibody incubation (30 min, dark), cells were washed, stained with live/dead dye (4 °C, 30 min, dark), fixed overnight at 4 °C, resuspended in Perm Buffer, and analyzed by flow cytometry.

### Western blotting

For protein extraction, cell pellets stored at − 80 °C were lysed in ice-cold RIPA buffer (supplemented with 1 μL PMSF per mL) with vortexing every 10 min for 30 min, then centrifuged (12 000 rpm, 15 min, 4 °C) to collect supernatants. Tumor tissues (3 samples per group pooled) were homogenized on ice in RIPA buffer (with 1 μL PMSF, protease inhibitor, and phosphatase inhibitor per mL) using bead milling (3 cycles: 20 s grinding, 10 min intervals), followed by centrifugation as above. Protein concentration was determined via BCA assay with a standard curve (0.25 mg/mL standard), and samples were adjusted to uniform concentration with ultrapure water and 5 × Loading buffer, denatured at 100 °C for 10 min, and stored at − 80 °C. For electrophoresis, 25 μg protein per sample was loaded; electrophoresis was performed at 60 V until reaching the stacking gel, then 120 V until completion. PVDF membranes were activated in methanol for 30 s, and transfer was conducted at 300 mA for 90 min on ice (filter paper-gel-membrane-filter paper sandwich in transfer buffer). Membranes were blocked for 90 min, washed 3 × 10 min with TBST, and incubated overnight at 4 °C with primary antibodies: Perforin (14,580-1-AP, Proteintech), Granzyme B (13588-1-AP, Proteintech), Beta Actin (66,009-1-Ig, Proteintech), AMOT (A8075, ABclonal), CD69 (A2045, ABclonal), ZAP70 (A9536, ABclonal; ab300398, Abcam), YAP1 (A19134, ABclonal), and Phospho-YAP1-S127 (AP1436, ABclonal). After TBST washes, membranes were incubated with secondary antibodies for 1 h at room temperature, washed again, and visualized with ECL substrate. Band intensities were quantified using ImageJ, normalized to Beta Actin.

### Immunofluorescence assay

Paraffin sections of tumors and mouse brain tissues were deparaffinized by sequential immersion in Xylene I, Xylene II, Anhydrous Ethanol I, and Anhydrous Ethanol II, then air-dried in a fume hood and rinsed with distilled water. Antigen retrieval and blocking were performed following standard protocols. Sections were incubated with primary antibodies overnight at 4 °C, including CD8A Rabbit mAb (A0663, Abclonal, China), CD45 Monoclonal antibody (60287-1-Ig, Abclonal, China), c-Fos Rabbit Polyclonal antibody (P23556, Yuantai Biotechnology, China), and CHAT Monoclonal Rabbit antibody (P17591, Yuantai Biotechnology, China). After washing with PBS, sections were incubated with fluorescent secondary antibodies at room temperature in the dark: FITC-conjugated goat anti-mouse IgG (H + L) (AS001, Abclonal, China) and ABflo 555-conjugated goat anti-rabbit IgG (H + L) (AS058, Abclonal, China). For nuclear counterstaining, sections were washed 3 times with PBS on a decolorizing shaker, drained slightly, and incubated with DAPI staining solution for 10 min at room temperature in the dark. After another 3 washes with PBS, sections were air-dried slightly and mounted with anti-fluorescence quenching mounting medium. Images were captured using an inverted fluorescence microscope.

### ELISA assay

Serum and tumor tissue supernatants were stored at − 80 °C until use. Mouse IL-2 (EMC002.48, NeoBioscience, China), IFN-γ (EMC101g.48, NeoBioscience, China), ACh (E-EL-0081c, Elabscience, China), EPI (E-EL-0045c, Elabscience, China), and NA/NE (E-EL-0047c, Elabscience, China) ELISA kits were equilibrated to room temperature for 20 min (from 2 to 8 °C storage). Assays followed manufacturers’ instructions. All serum samples were diluted at a ratio of 1:2 using the assay buffer provided with the kit. Supernatants of tumor tissues were diluted at a ratio of 1:5 to ensure that the detected concentrations fell within the linear range of the standard curve. All samples in the same experiment were subjected to the identical dilution factor to eliminate inter-sample variations. The final results presented were calculated by multiplying the measured values by the corresponding dilution factors. 100 μL of standards or diluted samples was added to pre-coated 96-well plates, incubated at 37 °C in the dark for 60 min, then washed 6 times. 100 μL HRP-conjugated detection antibody was added, incubated for 30 min (37 °C, dark), and washed 3 times. 100 μL TMB substrate was added for 15 min (37 °C, dark); 50 μL stop solution terminated the reaction. Absorbance at 450 nm was read within 30 min, and target concentrations were calculated via standard curves.

### Transcriptome sequencing

Total RNA was extracted from 50 to 100 mg tumor tissues: tissues were fully ground in a liquid nitrogen-precooled mortar, homogenized with 1 mL Trizol lysis buffer, and RNA was isolated via chloroform phase separation, isopropanol precipitation, and 75% ethanol washing. RNA was dissolved in RNase-free water; purity was detected by Nanodrop and integrity verified by Agilent Bioanalyzer. To minimize batch effects, all RNA samples were processed simultaneously by the same operator. Library construction and sequencing were performed in a single batch on the Illumina Novoseq 6000 platform (Shanghai Majorbio Bio-pharm Technology Co., Ltd.). mRNA was enriched with oligo beads, fragmented, reverse-transcribed into cDNA, and linked to Illumina adapters to construct libraries. Sequencing depth was ≥ 30 million reads per sample. Raw sequencing data were normalized using the sva R package to remove batch-specific variation and quality-controlled with FastQC and low-quality sequences filtered by Trimmomatic. Additionally, we included three biological replicates per group and used DESeq2 to account for residual batch effects during differential gene analysis.

Differential gene analysis was performed with DESeq2: a negative binomial distribution model based on sample conditions was constructed; after dispersion estimation and Wald test, significantly differentially expressed genes (DEGs) were screened with |FC|> 1.2 and *P* < 0.05. Functional annotation of DEGs was conducted using R package clusterProfiler (v3.12.0): Gene Ontology (GO) enrichment was analyzed via the enrichGO function (statistical test against species-specific databases); Kyoto Encyclopedia of Genes and Genomes (KEGG) pathway mapping was performed via the enrichKEGG function (organism parameters specified, *P*-values adjusted by Benjamini–Hochberg method). Significantly enriched results (*P* < 0.05) were visualized as bar charts, bubble charts, and gene-pathway networks. Batch effects were controlled and tool versions unified throughout the experiment to ensure reproducibility.

### Bioinformatics and survival analysis

The prognostic value of NGF mRNA expression in breast cancer was analyzed using two independent online platforms. First, the Gene Expression Profiling Interactive Analysis (GEPIA; http://gepia.cancer-pku.cn/) database was used to generate recurrence-free survival (RFS) curves for breast cancer patients based on TCGA data. Patients were stratified into high- and low-expression groups using the median NGF expression level as the cutoff, with the hazard ratio (HR) and Log-rank P-value automatically calculated. To further verify the prognostic significance in the triple-negative breast cancer (TNBC) subtype, we additionally employed the Kaplan–Meier Plotter tool (https://kmplot.com/analysis/). The analysis was restricted to TNBC patients by setting the parameters to “ER negative,” “PR negative,” and “HER2 negative” according to IHC or array criteria, with the probe set 206814_at selected for NGF expression quantification. Survival curves were plotted with 95% confidence intervals (CIs), and statistical significance was assessed using the Log-rank test.

### Real-time PCR assay

Cell/tissue samples were lysed, centrifuged, and RNA extracted via chloroform phase separation, isopropanol precipitation, and 75% ethanol washing. RNA was dissolved in nuclease-free water and quantified by UV spectroscopy. RNA was denatured at 65 °C, reverse-transcribed at 37 °C for 15 min, and enzyme-inactivated at 98 °C for 5 min (stored at − 20 °C). Diluted primers were used for real-time PCR (prepared on ice) with cycling conditions: 95 °C for 60 s, then 40 cycles of 95 °C (15 s), 60 °C (15 s), 72 °C (45 s). Fluorescence data were analyzed with StepOne v2.1 software (9 replicates per target). Relative expression was calculated using the 2^−^∆∆CT method (∆CT = target CT—reference CT; ∆∆CT = experimental ∆CT—model group ∆CT).

### Cell functional assays

For the cell invasion assay, transwell chambers (in 24-well plates) were coated with Matrigel (1:8 dilution in serum-free medium, 50 μL/chamber) and solidified at 37 °C for 30 min. Log-phase 4T1 and MDA-MB-231 cells (5 × 10^5^ cells/mL) were pretreated with serum-free medium containing β-NGF (200 ng/mL) or VP (2 μm) for 24 h (control: serum-free medium alone). 100 μL cell suspension (5 × 10^4^cells) was added to the upper chamber, and 600 μL medium with 20% FBS to the lower chamber. After 24 h of culture (37 °C, 5% CO_2_), non-invading cells were removed; invading cells were fixed (4% paraformaldehyde, 30 min), stained (0.1% crystal violet, 20 min), and counted in 6 random fields under a microscope.

For the cell migration assay, cells were seeded in 6-well plates, cultured to 90% confluency, and scratched with a 200 μL pipette tip. After washing with pre-warmed PBS, initial scratch widths were imaged. Cells were then cultured in serum-free medium with β-NGF (200 ng/mL) or VP (2 μm) for 24 h (control: serum-free medium alone), and scratches were re-imaged at the same positions. Migration rate was quantified using ImageJ software. The scratch width was measured at three random positions per well at 0 h and 24 h. Migration rate (%) = [(Initial scratch width—Final scratch width) / Initial scratch width] × 100%. Three independent experiments were performed, with three technical replicates per group.

For the colony formation assay, 4T1 and MDA-MB-231 cells pretreated with β-NGF (200 ng/mL) for 24 h were seeded in 6-well plates (400 cells/well, 2 mL medium) and cultured for 2 weeks (37 °C, 5% CO_2_), with medium containing β-NGF or VP refreshed every 3 days. Colonies were fixed (4% paraformaldehyde, 30 min), stained (0.1% crystal violet, 20 min), and colonies > 0.3 mm (≥ 50 cells) were counted. Colony formation rate (%) = (number of colonies/number of seeded cells) × 100.

### Statistical analysis

All data were tested for normality using the Shapiro–Wilk test prior to statistical analysis. Data conforming to a normal distribution were analyzed by one-way ANOVA, with the LSD test applied for homogeneous variances and the Games-Howell test for heterogeneous variances. Data failing to meet the normality assumption were analyzed using nonparametric tests followed by Dunn’s post-hoc test. All statistical analyses were performed using SPSS 25.0 software, with *P* < 0.05 considered statistically significant. Statistical graphs were generated using GraphPad Prism 7.0 software.

## Results

### Screening of effective EA acupoints and intensity for TNBC

Based on previous neurobiological mechanisms and clinical evidence of EA in immune regulation, this study selected ST36 (Zusanli) and ST25 (Tianshu) as intervention acupoints. Multiple studies have shown that these two acupoints exert significant anti-inflammatory and immune regulatory effects through different neuro-immune regulatory pathways [13–17].

**Fig. 1:**
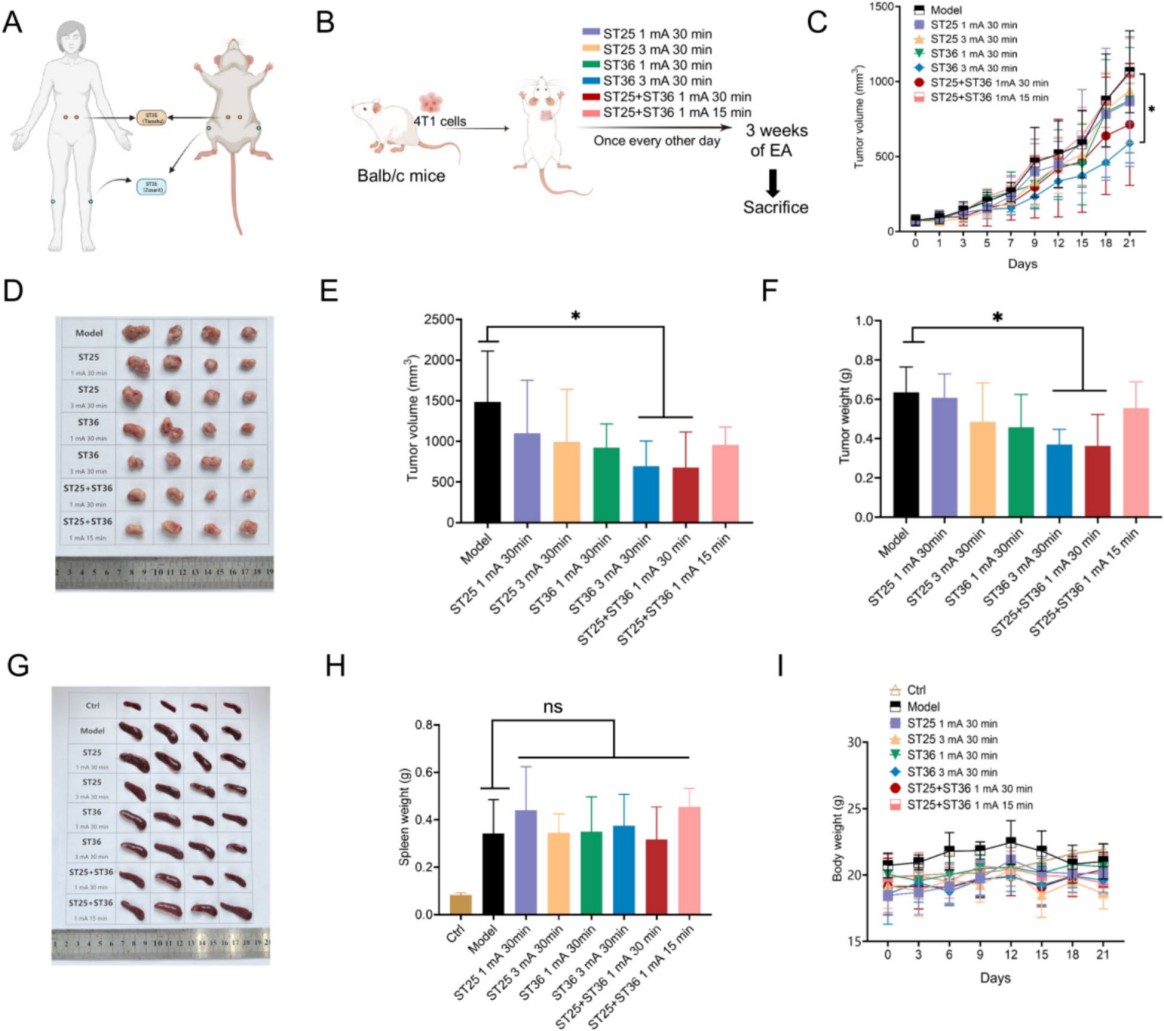
Effects of different acupoints and intervention intensities on TNBC mice. **A** Schematic representation of the anatomical locations of ST36 and ST25 acupoints in mice and humans. Murine ST36 is located on the posterolateral aspect of the knee joint, approximately 2 mm inferior to the head of the fibula; murine ST25 is located on the abdomen, 3 mm lateral to the midline; human ST36 is located 3 cun inferior to the knee joint, in the depression between the tibia and fibula; human ST25 is located on the mid-abdomen, 2 cun lateral to the center of the umbilicus. Cun is a traditional Chinese anatomical measurement unit; 1 cun ≈ 20–25 mm (0.02–0.025 m) in adult humans. **B** Schematic diagram of model establishment and administration (n = 6). **C** Tumor growth trend in each group. **D** Representative images of tumors in each group. **E** Tumor volume. **F** Tumor weight. **G** Representative images of spleens in each group. **H** Spleen weight of mice in each group. **I** Body weight change trend of mice in each group. The Ctrl group consisted of healthy, tumor-free Balb/c mice that received neither tumor cell inoculation nor EA treatment. *P < 0.05, *ns*, no significance, *n* = 5

A TNBC xenograft model was successfully established by orthotopic injection of the murine TNBC cell line 4T1. Mice in the control group (Ctrl) were maintained under identical environmental conditions without tumor induction or any therapeutic intervention to serve as a physiological baseline. Tumor-bearing mice were randomly divided into the following groups: model control group, ST25 (1 mA, 30 min) group, ST25 (3 mA, 30 min) group, ST36 (1 mA, 30 min) group, ST36 (3 mA, 30 min) group, ST25 + ST36 (1 mA, 30 min) group, and ST25 + ST36 (1 mA, 15 min) group (Fig. 1A–B). A HANS 200A Korean-style EA instrument (Nanjing Jisheng Medical Co., Ltd.) was used with a 2/15 Hz, administered every other day for 3 consecutive weeks. After 3 weeks of intervention, it was found that ST36 (3 mA, 30 min) and ST25 + ST36 (1 mA, 30 min) groups significantly inhibited tumor growth and weight in TNBC mice (*P* < 0.05) (Figs. 1C–F). Except for the model control group, there were no significant differences in spleen size or weight among the treatment groups (Figs. 1G–H). No significant differences in body weight were observed among the groups during the treatment period (Fig. 1I).

### EA modulates immune cell subsets in immune microenvironment of TNBC mice

**Fig. 2:**
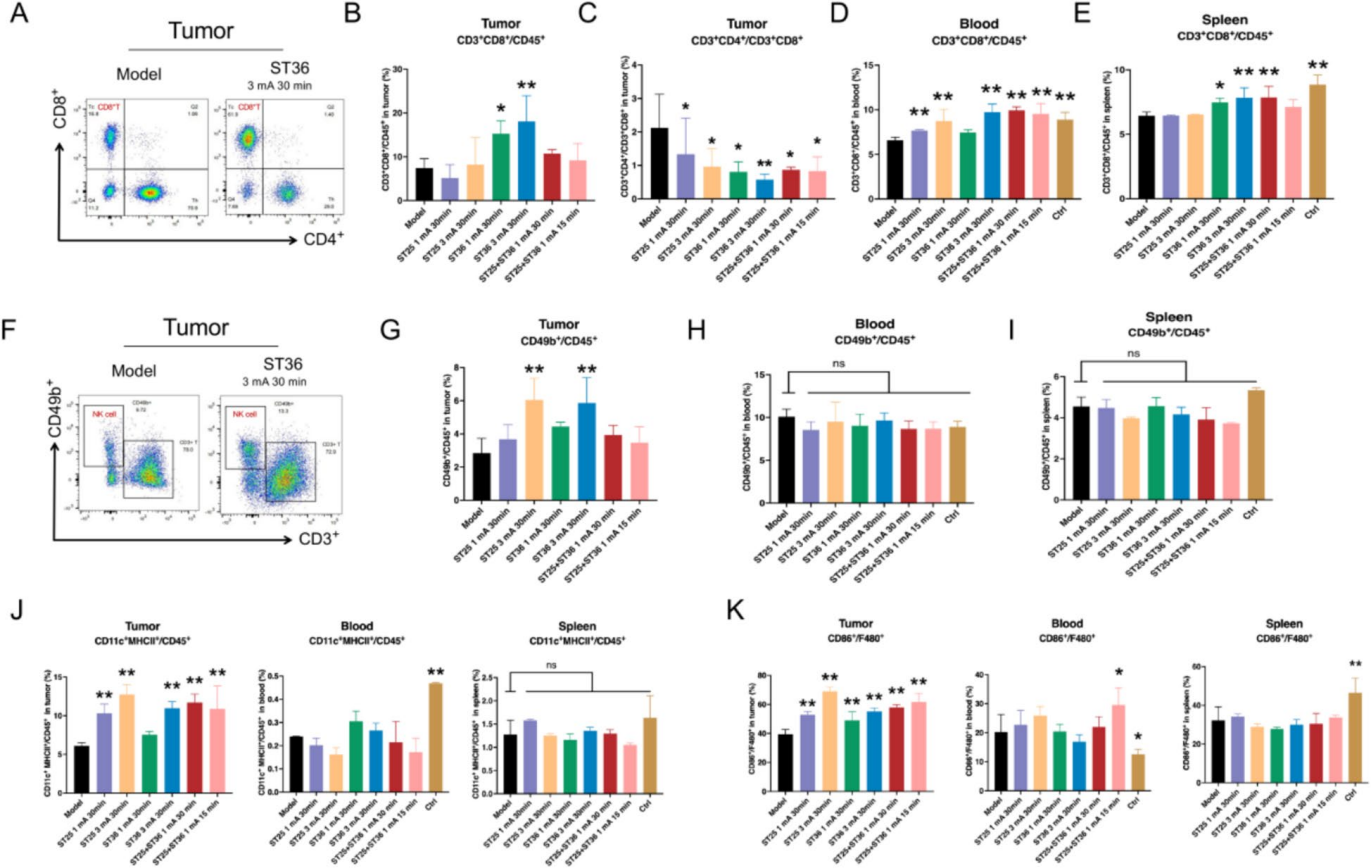
EA preferentially expands intratumoral CD8^+^ T cells and remodels the tumor immune microenvironment in TNBC mice. **A**–**B** Representative flow cytometry plots and statistical analysis of CD8^+^ T cell infiltration in tumor tissues. **C** The ratio of CD4^+^/CD8^+^ T cells in tumor tissues across different groups. **D**–**E** Proportions of CD8^+^ T cells in the peripheral blood (**D**) and spleens (**E**). **F**–**G** Representative flow cytometry plots and statistical analysis of NK cell infiltration (CD49b^+^) in tumor tissues. **H**–**I** Percentages of NK cells in the peripheral blood (**H**) and spleens (**I**). **J** Quantitative analysis of DC infiltration (CD11c^+^ MHCII^+^) in tumor tissues, peripheral blood, and spleens. **K** The proportion of M1-type macrophages (CD86^+^F4/80^+^) in tumor tissues, peripheral blood, and spleens. The Ctrl group consisted of healthy, tumor-free Balb/c mice that received neither tumor cell inoculation nor EA treatment. *P < 0.05, **P < 0.01, *ns:* no significance, *n* = 5

To systematically evaluate the effect of EA intervention on the immune microenvironment, flow cytometry was used to detect changes in immune cell subsets in tumor tissues, peripheral blood, and spleens of the TNBC mouse model. The abundance of tumor-infiltrating CD8^+^ T cells and the CD4^+^/CD8^+^ ratio serve as pivotal benchmarks for assessing immunotherapeutic efficacy [18]. Our findings demonstrated that EA intervention at ST36 (1 mA, 30 min) and ST36 (3 mA, 30 min) groups markedly boosted CD8^+^ T cell populations within the tumor microenvironment (*P* < 0.01, *P* < 0.05) (Fig. 2A–B), while other EA groups also exerted varying degrees of influence on the intratumoral CD4^+^/CD8^+^ ratio (*P* < 0.01, *P* < 0.05) (Fig. 2C). In the blood, a heightened proportion of CD8^+^ T cells was observed across all intervention groups except for the ST36 (1 mA, 30 min) group when compared to the model controls (Fig. 2D). Similarly, splenic CD8^+^ T cell frequencies were upregulated in the ST36 (1 mA, 30 min), ST36 (3 mA, 30 min) and the ST25 + ST36 (1 mA, 30 min) groups (*P* < 0.01, *P* < 0.05) (Fig. 2E). Overall, the ST36 (3 mA, 30 min) regimen yielded the most profound immunomodulatory effects.

Subsequent subset analysis revealed that at the innate immune level, both ST36 and ST25 treatments at 3 mA significantly enriched NK cell infiltration within tumors (*P* < 0.01) (Fig. 2F–G), although systemic NK cell counts in the blood and spleen remained largely unaffected (Fig. 2H–I). At the adaptive immunity regulation level, except for the ST36 (1 mA, 30 min) group, the proportion of dendritic cells (DCs) in tumor tissues of other EA groups increased significantly, while no significant changes in DCs were observed in peripheral blood or spleens (*P* < 0.05) (Fig. 2J). All EA intervention groups demonstrated an increased proportion of M1-type macrophages within tumor tissues, an effect that was largely absent in the peripheral circulation or splenic macrophage populations (*P* < 0.01, *P* < 0.05) (Fig. 2K), with the notable exception of the ST25 + ST36 (1 mA, 15 min) group, which showed a similar trend in the peripheral blood.

Collectively, these findings delineate a multifaceted immunomodulatory profile elicited by EA intervention, encompassing coordinated enhancements in both innate and adaptive immune compartments predominantly within the TME. Notably, CD8^+^ T cell enrichment emerged as the most consistent immunological change, particularly within the tumor microenvironment where the ST36 (3 mA, 30 min) regimen achieved the most pronounced effects. The convergent elevation of intratumoral CD8^+^ T cell abundance, peripheral CD8^+^ T cell mobilization, and splenic CD8^+^ T cell expansion collectively suggest that CD8^+^ cytotoxic T lymphocytes may represent the central immune effector population driving EA-mediated anti-tumor immunity in TNBC.

### EA inhibits tumor growth and proliferation but not lung metastasis in TNBC mice

Through the analysis of the above series of experimental results, we clearly observed that EA stimulation at ST36 (3 mA, 30 min) exerted a significant inhibitory effect on the tumor growth process of the TNBC mouse model. Meanwhile, compared with other EA groups, EA at ST36 (3 mA, 30 min) exerted the most positive regulatory effect on the immune function of TNBC mice. Based on this key finding, the research team selected ST36 as the therapeutic acupoint, with a fixed current intensity of 3 mA and an intervention duration of 30 min, to further conduct in-depth follow-up experiments.

**Fig. 3:**
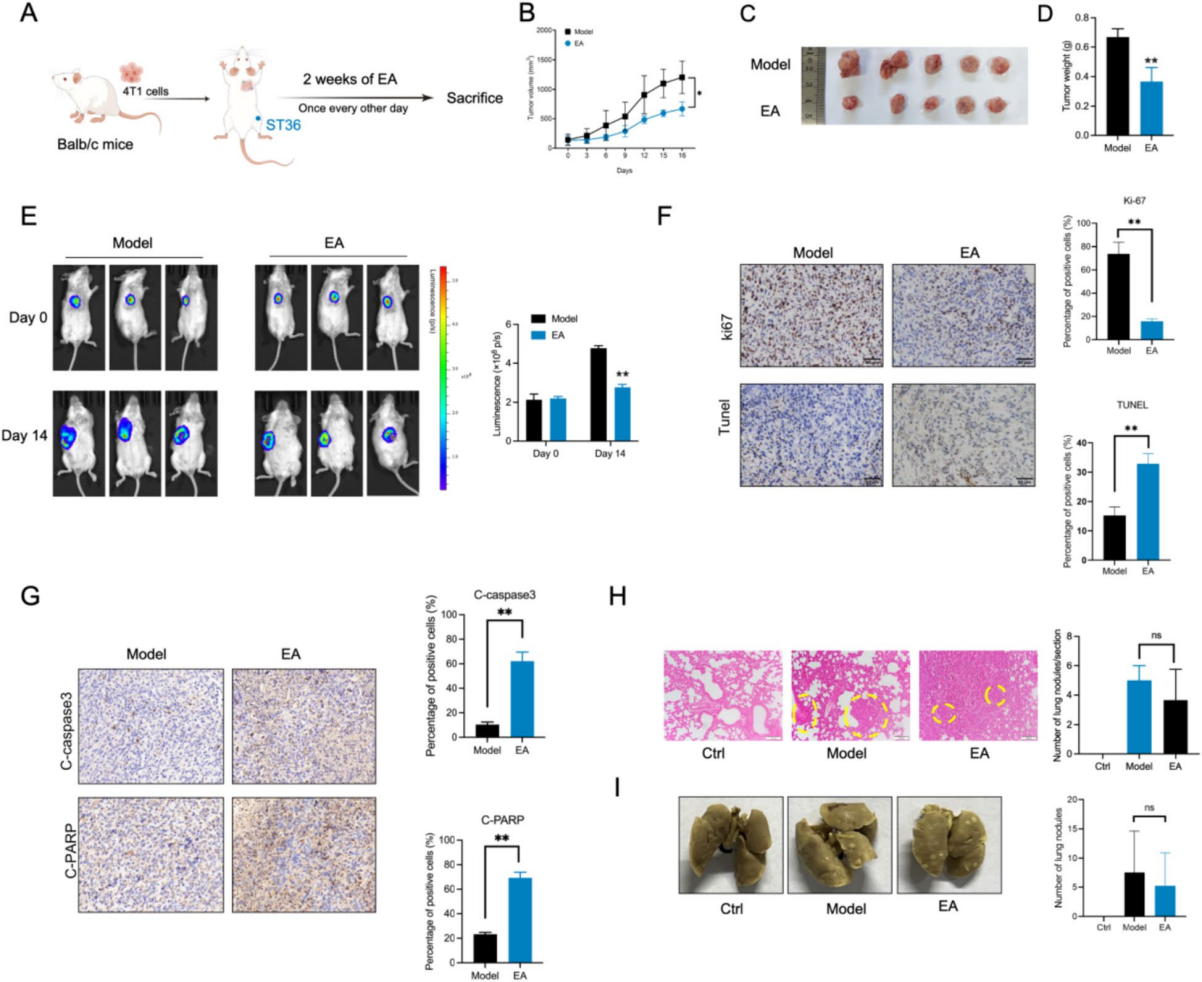
Effects of EA at ST36 (Zusanli) in TNBC mice. **A** Schematic diagram of model establishment and administration. **B** Tumor growth trend in each group. **C** Representative images of tumors in each group. **D** Tumor weight in each group. **E** In vivo imaging of tumor size before treatment and 2 weeks after treatment in each group. **F** Expression of Ki-67 and Tunel in tumor tissues of each group. **G** Expression of Cleaved-caspase 3 and Cleaved-PARP in tumor tissues of each group. **H** HE staining to observe lung metastasis in each group. **I** Number of lung nodules in each group. *P < 0.05, **P < 0.01, *ns:* no significance, *n* = 5

After 2 weeks of treatment, the tumor volume of mice in the EA group was significantly lower than that in the model control group (*P* < 0.05). This result was consistent with the data obtained in the first part of the study, verifying the stability and reproducibility of the tumor growth inhibitory effect of EA at ST36 (Fig. 3A–D). To more intuitively observe the dynamic changes of tumors in the living state, in vivo imaging technology was used to monitor the tumor size of mice in each group before treatment and after 2 weeks of treatment. The results clearly showed that the tumor volume of mice in the EA group was significantly smaller than that in the model control group (Fig. 3E), further confirming the anti-tumor effect of EA intervention.

On the basis of macroscopic comparison of tumor volume and weight, immunohistochemical staining was used to detect the expression of the proliferation marker Ki-67 and apoptosis marker Tunel in tumor tissues. The results showed that the expression level of the proliferation marker Ki-67 in tumor tissues of mice in the EA group was significantly reduced, while the expression level of the apoptosis marker Tunel was significantly increased (*P* < 0.01), indicating that EA at ST36 could inhibit tumor cell proliferation and induce tumor cell apoptosis (Fig. 3F). To further validate the pro-apoptotic effect of EA on TNBC in vivo, we assessed the expression of classic apoptosis-related markers. IHC analysis demonstrated that EA treatment led to a significant increase in the positive staining percentages of Cleaved Caspase-3 and Cleaved PARP compared to the Model group (*P* < 0.01) (Fig. 3G). These findings, consistent with our tumor growth data, confirm that EA effectively triggers the apoptotic cascade in TNBC tissues. Considering that the 4T1 cell line is a TNBC cell line with high lung metastasis potential, the lung metastasis of mice was systematically observed by HE staining and lung nodule count. However, the experimental results showed that EA intervention did not exhibit a significant inhibitory effect on the lung metastasis process of TNBC mice (Fig. 3H–I).

### EA activates vagal nerve conduction and regulates neurotransmitter levels in TNBC mice

**Fig. 4:**
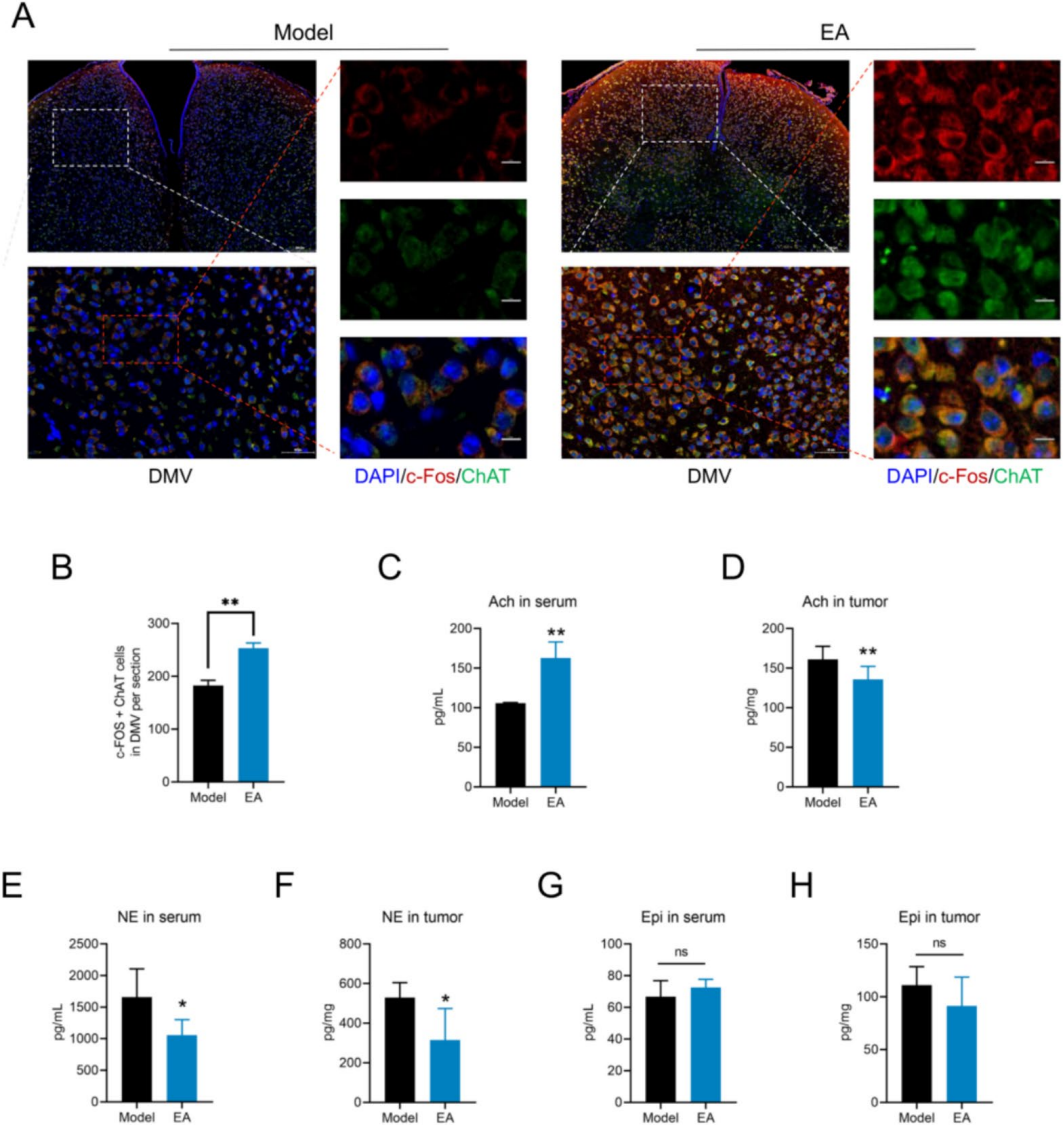
Effects of EA at ST36 (Zusanli) on nerve conduction and neurotransmitters in TNBC mice. **A**–**B** Immunofluorescence double labeling to observe the number of c-Fos^+^/ChAT^+^ double-positive neurons in the dorsal motor nucleus of the vagus (DMV); c-Fos (red) marks activated neurons, and ChAT (green) marks cholinergic vagal neurons. **C**–**D** ELISA detection of acetylcholine (Ach) content in serum and tumor tissues. **E**–**F** ELISA detection of norepinephrine (NE) content in serum and tumor tissues. **G**–**H** ELISA detection of epinephrine (Epi) content in serum and tumor tissues. *P < 0.05, **P < 0.01, *ns:* no significance, *n* = 5

Multiple studies have shown that EA at ST36 can drive vagus nerve-adrenergic nerve conduction and regulate the release of neurotransmitters such as norepinephrine (NE), epinephrine (EPI), and acetylcholine (Ach) [16, 19]. To observe the effect of EA on vagal nerve conduction in TNBC mice, immunofluorescence was used to detect vagal nerve conduction in the dorsal motor nucleus of the vagus (DMV) region of the mouse brain. The results showed that after EA, the co-expression of c-Fos and choline acetyltransferase (ChAT) in the DMV region of TNBC mice was significantly increased, suggesting that EA could drive vagal nerve conduction in TNBC mice (*P* < 0.01) (Fig. 4A–B). The vagus nerve is the tenth cranial nerve, which originates from the brainstem, passes through the neck, and innervates organs in the chest and abdomen, and is an important component of the parasympathetic nervous system [20]. Vagus nerve stimulation (VNS) has been shown to regulate neural activity and the conduction and functional activities of the neuroendocrine-immune axis [21]. VNS effectively inhibits excessive sympathetic excitation and reestablishes the dynamic balance of the autonomic nervous system by activating parasympathetic fibers of the vagus nerve; studies have shown that acetylcholine released from vagal nerve terminals can act on M-type cholinergic receptors in sympathetic nerve terminals, reducing NE release from sympathetic nerve terminals; VNS can reduce the level of sympathetic hyperactivity markers (such as NE) by prolonging the duration of vagal nerve action potentials [22, 23]. ELISA was used to quantitatively analyze neurotransmitter levels in the peripheral blood and tumor tissues of mice. The results showed that in peripheral blood, EA intervention significantly upregulated the expression level of ACh (*P* < 0.01) (Fig. 4C); in contrast, the ACh content in tumor tissues showed a significant downward trend (*P* < 0.01) (Fig. 4D). For NE, after EA intervention, the NE levels in both peripheral blood and tumor tissues decreased significantly (*P* < 0.05) (Fig. 4E–F). However, no statistically significant changes in Epi expression were observed in either peripheral blood or tumor tissue samples (Fig. 4G–H). The consistent reduction of NE in both systemic circulation and the tumor microenvironment suggests that EA mitigates tumor growth by alleviating sympathetic hyperactivation and restoring autonomic homeostasis. These findings demonstrate that the anti-tumor efficacy of EA is primarily mediated by the downregulation of NE levels.

### EA enhances CD8^+^ T cell tumor infiltration and effector molecule levels in TNBC

**Fig. 5:**
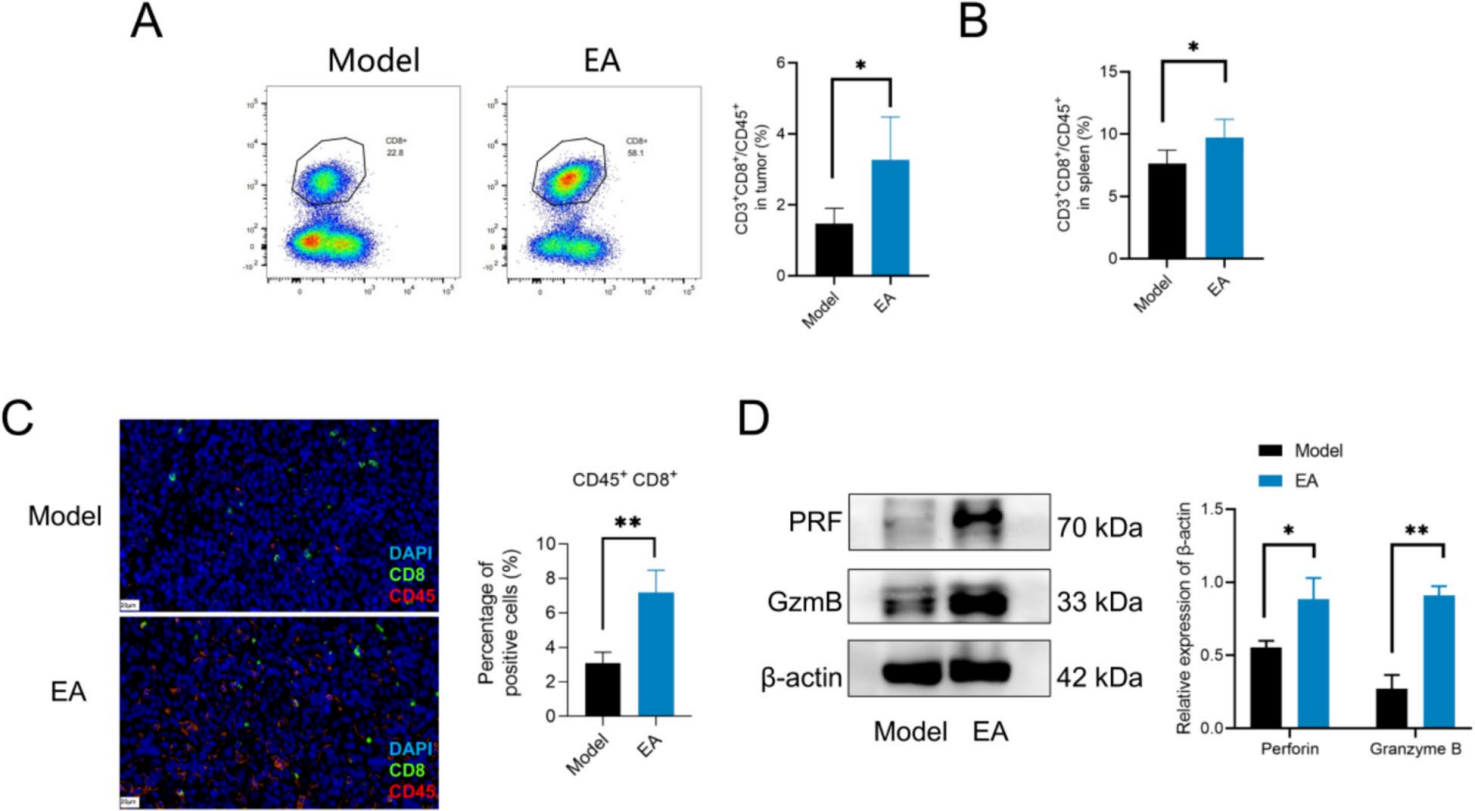
EA at ST36 (Zusanli) upregulates CD8^+^ T cell infiltration and effector molecule expression. **A** Representative flow cytometry plots and statistical analysis of CD8^+^ T cell infiltration within tumor tissues. **B** Flow cytometry analysis of CD8^+^ T cell proportions in the spleens of mice. **C** Representative immunofluorescence images showing CD8^+^ T cell infiltration in tumor tissues (Scale bar = 20 μm). CD45^+^ cells are labeled in red, CD8^+^ cells in green, and nuclei are stained with DAPI (blue). **D** Western blot analysis and quantification of perforin (PRF) and granzyme B (GzmB) protein levels in tumor tissues. *P < 0.05, **P < 0.01, *n* = 5

Flow cytometry showed that compared with the model control group, the proportion of CD8^+^ T cells in tumor tissues and spleens of mice in the EA group was significantly increased (*P* < 0.05) (Fig. 5A–B), which was consistent with the trend of previous experimental results. Immunofluorescence analysis further confirmed that EA stimulation at ST36 significantly increased the infiltration of CD8^+^ T cells in TNBC tumor tissues (*P* < 0.01) (Fig. 5C).

Perforin (PRF) and granzyme B (GzmB) are core effector molecules of CD8^+^ T cell-mediated anti-tumor immune responses [24]. Among them, PRF promotes GzmB entry into target cells by forming transmembrane pores, and GzmB induces tumor cell death through multiple pathways, such as activating the caspase cascade, disrupting DNA repair proteins, and inducing mitochondrial apoptosis [25, 26]. Western blot results showed that compared with the model control group, the protein expression levels of PRF and GzmB in tumor tissues of the EA group were significantly upregulated (*P* < 0.05, *P* < 0.01) (Fig. 5D). These experimental data indicate that EA stimulation at ST36 may exert an inhibitory effect on TNBC growth by enhancing the tumor infiltration ability of CD8^+^ T cells and promoting the expression of their effector factors.

### EA potentiates with αPD-L1 to inhibit TNBC via CD8^+^ T cell-mediated anti-tumor immunity

Tumor cells bind to programmed cell death protein 1 (PD-1) on the surface of CD8^+^ T cells through programmed cell death ligand 1 (PD-L1), inhibiting the activation, proliferation, and effector function of T cells. This interaction significantly reduces the release of cytotoxic molecules (such as PRF and GzmB) by CD8^+^ T cells and inhibits the production of pro-inflammatory factors such as IFN-γ and interleukin (IL)-2. Immunotherapeutic drugs represented by PD-L1 inhibitors inhibit tumor progression by restoring the killing ability of CD8^+^ T cells, but their efficacy depends on pre-existing CD8^+^ T cell infiltration [27, 28].

**Fig. 6:**
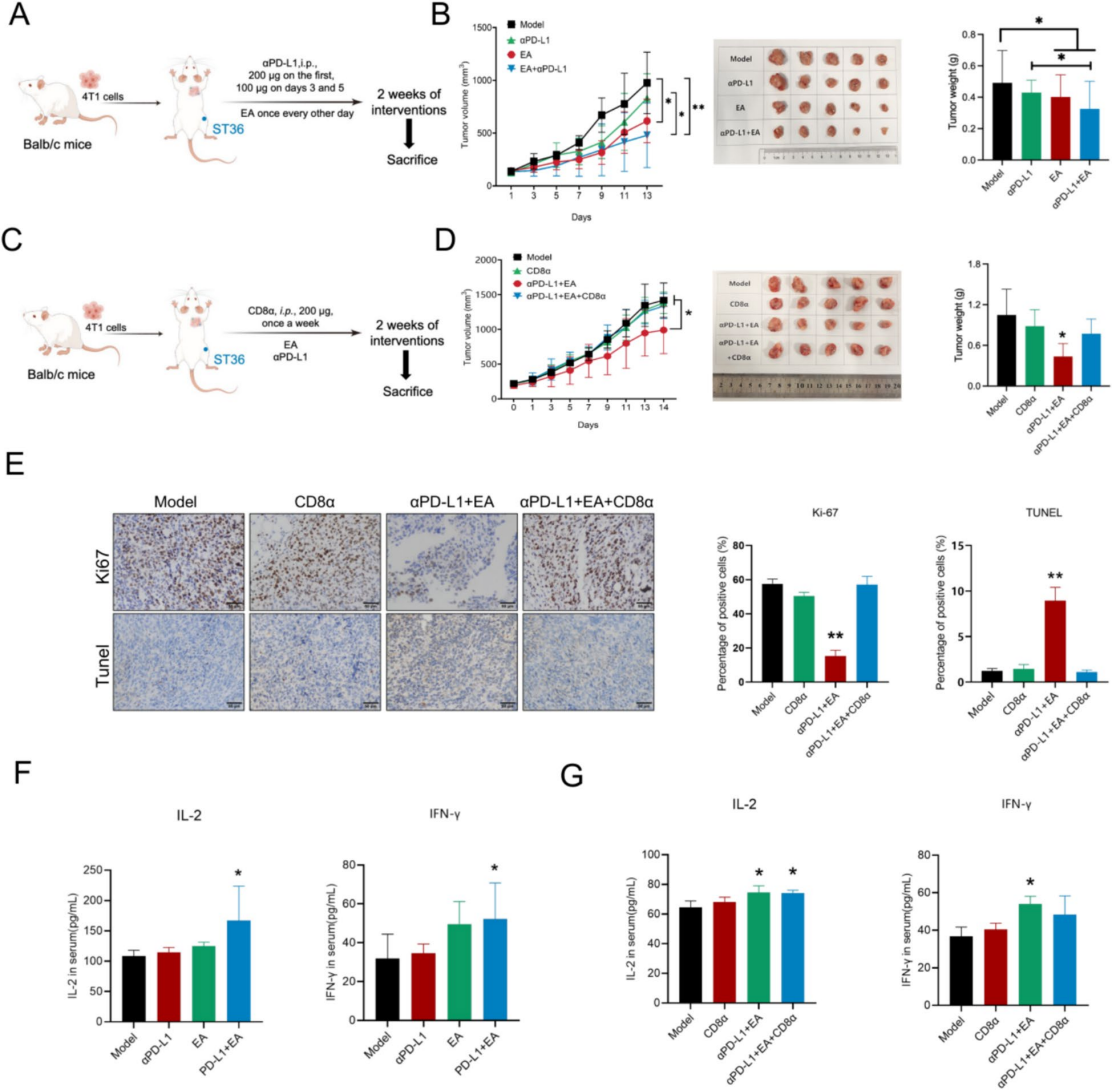
EA enhances the therapeutic efficacy of αPD-L1 in TNBC dependent on CD8^+^ T cells. **A** Schematic illustration of the experimental design. The αPD-L1 was administered *intraperitoneally (i.p.)* at an initial dose of 200 μg, followed by 100 μg on day 3 and day 5. EA treatment was performed every other day; when EA and αPD-L1 administration were scheduled on the same day, EA was conducted 2 h prior to antibody injection. **B** Representative tumor images, tumor growth curves, and tumor weights in each group. **C** Schematic illustration of the CD8^+^ T cell depletion experiment. Anti-CD8α antibody (200 μg per injection) was administered *i.p.* 2 days before the first EA/αPD-L1 treatment, once weekly for 3 consecutive weeks. EA and αPD-L1 were administered using the same regimen as described in Fig. 6A. **D** Representative tumor images, tumor growth curves, and tumor weights in each group after CD8^+^ T cell depletion. **E** Expression of Ki-67 and TUNEL in tumor tissues. **F**–**G** ELISA analysis of interleukin-2 (IL-2) and interferon-γ (IFN-γ) levels in the serum. *P < 0.05, **P < 0.01, *n* = 5

Therefore, after confirming that EA at ST36 could enhance CD8^+^ T cells in TNBC mice, we further evaluated its effect with αPD-L1. Experiments showed that the tumor volume of the EA combined with αPD-L1 group was significantly smaller than that of the model control group (*P* < 0.01), and there was a significant difference compared with the αPD-L1 monotherapy group (*P* < 0.05) (Fig. 6A–B). To explore the role of CD8^+^ T cells in this effect, continuous depletion of CD8^+^ T cells was achieved by injecting CD8α antibody every other day. The results showed that after CD8^+^ T cell depletion, the effect of EA enhancing αPD-L1-mediated tumor growth inhibition completely disappeared (Fig. 6C–D); the changes in Ki-67 and Tunel expression in tumor tissues also showed the same trend (Fig. 6E), suggesting that CD8^+^ T cells are key mediators of EA synergizing with αPD-L1.

IL-2 and IFN-γ are key cytokines for CD8^+^ T cells to exert anti-tumor effects. Among them, IL-2 drives the proliferation of CD8^+^ T cells, maintains memory T cells, and inhibits regulatory T cells (Tregs), while IFN-γ exerts its effects by enhancing tumor cell antigen presentation, reprogramming the immune microenvironment, inhibiting angiogenesis, and interfering with tumor metabolism [29, 30]. Quantitative analysis of mouse serum cytokine levels by ELISA showed that compared with the model control group and monotherapy groups, the expression levels of IL-2 and IFN-γ in the EA combined with αPD-L1 treatment group showed a significant upward trend (*P* < 0.05) (Fig. 6F). This result indicates that the combined intervention of EA and αPD-L1 can effectively activate the immune response mediated by CD8^+^ T cells, promoting them to secrete more effector cytokines, thereby enhancing the body’s anti-tumor immune capacity. Notably, after establishing a CD8^+^ T cell-specific depletion model, the inductive effect of this combination therapy on IFN-γ decreased (Fig. 6G), which mechanistically confirms that CD8^+^ T cells are key effector cells for EA synergizing with immune checkpoint inhibitors to exert therapeutic effects.

### Transcriptome analysis reveals EA modulates NGF and Hippo pathway

**Fig. 7:**
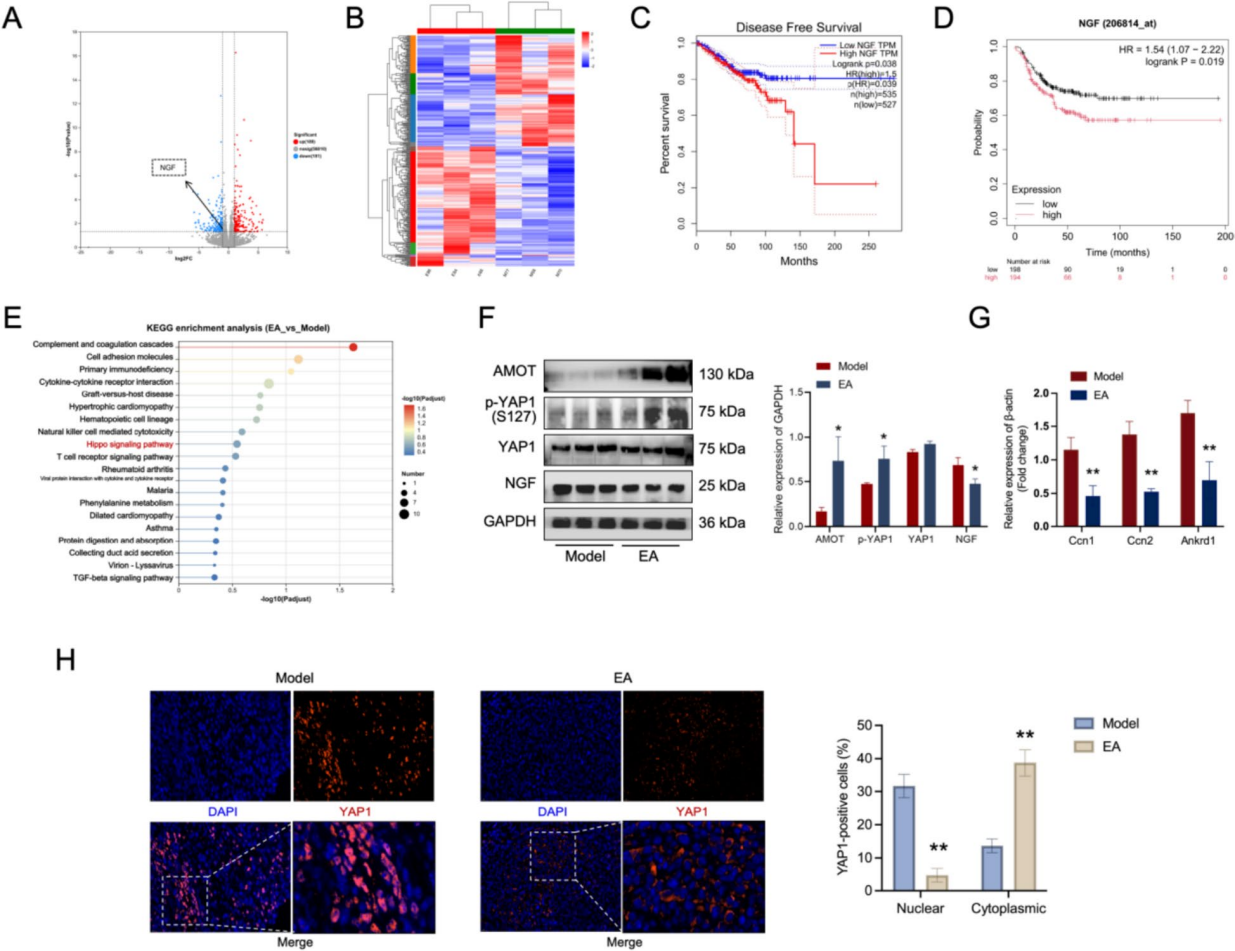
EA regulates NGF expression and activates the Hippo pathway via AMOT/YAP in TNBC. **A** Volcano plot of differentially expressed genes (DEGs). **B** Heatmap of DEGs. **C**–**D** Survival analysis of NGF expression on recurrence-free survival (RFS) of breast cancer patients. **E** Kyoto Encyclopedia of Genes and Genomes (KEGG) enrichment analysis. **F** Western blot detection of NGF, AMOT, YAP1 protein expression and YAP1 phosphorylation level at Ser127 (p-YAP1-S127) in tumor tissues, with three lanes per group representing individual biological replicates. **G** qPCR analysis of the relative mRNA levels of Ccn1, Ccn2, and Ankrd1 in the model and EA groups. **H** Immunofluorescence detection of YAP protein expression in tumor tissues. *P < 0.05, *n* = 3

To explore the mechanism of EA intervention in TNBC, transcriptome sequencing was used to identify differentially expressed genes and signaling pathways in tumor tissues between the model control group and EA group. The gene expression levels obtained by transcriptome sequencing were standardized, and differentially expressed genes were screened by comparing the two groups with the criteria of *P* < 0.05 and *Fold change (FC)* > 1.2. The results showed that EA downregulated 181 genes and upregulated 189 genes in tumor tissues of tumor-bearing mice (Figs. 7A–B, and supplementary Table 1). Among them, we noticed that EA could downregulate the gene expression of nerve growth factor (NGF) (*FC* = 0.483, *P* = 0.027).

NGF is the earliest discovered neurotrophic factor, which plays a core role in the nervous system by regulating the development, differentiation, survival, and regeneration of neurons. In recent years, studies have found that NGF is abnormally expressed in the tumor microenvironment, participating in tumor occurrence, development, and metastasis through multiple pathways [31], and can cause functional changes in immune cells such as T cells and M2-type macrophages [32].

To comprehensively evaluate the prognostic significance of NGF, survival analyses were performed across multiple clinical cohorts. Initially, using the GEPIA database, we assessed the association between NGF expression and recurrence-free survival (RFS) in a general breast cancer population. Patients were stratified into high- and low-expression groups based on median NGF levels. Kaplan–Meier analysis revealed that elevated NGF expression was significantly associated with poorer RFS (*HR* = 1.5, *P* = 0.038; Fig. 7C). To further validate these findings specifically in the triple-negative breast cancer (TNBC) subtype, we utilized the Kaplan–Meier Plotter database. By focusing on a cohort of TNBC patients within the 206814_at dataset, we observed a consistent trend: patients in the low-NGF expression group exhibited significantly superior RFS compared to the high-expression group (*HR* = 1.54, *P* = 0.019; Fig. 7D). Taken together, these results from independent datasets robustly demonstrate that high NGF expression is a negative prognostic indicator for TNBC patients.

KEGG enrichment analysis was performed on differentially expressed genes to identify signaling pathways significantly regulated by EA in TNBC. With *P* value < 0.05 and *Q* value < 0.05 as the significance thresholds, a scatter plot was drawn for the 20 signaling pathways with the largest number of genes. As shown in Fig. 7E, the involved signaling pathways include complement and coagulation cascades, cell adhesion molecules, cytokine-cytokine receptor interaction, hematopoietic cell lineage, NK cell-mediated cytotoxicity, Hippo signaling pathway, T cell receptor signaling pathway, TGF-β signaling pathway, etc.

The Hippo signaling pathway is a highly conserved signal transduction pathway that controls organ size and tissue homeostasis by regulating cell proliferation, apoptosis, and stem cell self-renewal. Its activation or inactivation state is closely related to the proliferation and metastasis of cancer cells [33, 34]. We found that EA could regulate the signal transduction of the Hippo pathway (*P* = 0.012) and regulate the expression of genes in this pathway, such as angiomotin (AMOT; *FC* = 2.001, *P* = 0.001), bone morphogenetic protein receptor type 1B (BMPR1B; *FC* = 0.448, *P* = 0.035), alpha-fetoprotein (AFP; *FC* = 0.170, *P* = 0.002), growth differentiation factor 6 (GDF6; *FC* = 2.114, *P* = 0.018), Wnt family member 4 (WNT4; *FC* = 2.876, *P* = 0.019), and bone morphogenetic protein 5 (BMP5; *FC* = 0.060, *P* < 0.011).

Among them, AMOT is an upstream regulator of the Hippo pathway. It regulates the nucleocytoplasmic localization of Yes-associated protein (YAP) by directly binding to YAP, promoting its retention in the cytoplasm or adhesion junctions, and plays a role in inhibiting the occurrence and development of malignant tumors [35, 36] and reducing the activity of breast cancer stem cells [37]. YAP is an important transcriptional co-activator, and its abnormal activation is closely related to the occurrence and development of various cancers, making it a core effector of the Hippo pathway [38]. When the Hippo pathway is activated, YAP binds to 14-3-3 proteins in the cytoplasm and is anchored in the cytoplasm, unable to enter the nucleus; when the Hippo pathway is inactivated, YAP enters the nucleus in a non-phosphorylated form and exerts pro-cancer effects [39]. Therefore, we hypothesized that EA might upregulate AMOT, activate the Hippo pathway, promote the phosphorylation of YAP protein at the S127 site, and retain it in the cytoplasm, thereby inhibiting tumor progression; this might be related to the inhibitory effect of EA on NGF.

To verify this hypothesis, Western blot experiments were performed to detect the protein expression of NGF, AMOT, YAP1, and p-YAP1 in the model control group and EA group. The results showed that NGF in tumor tissues of the EA group was significantly decreased, the protein expression of AMOT was significantly increased, the expression of total YAP1 protein showed no obvious change, but the phosphorylation level at its S127 site was significantly increased (Fig. 7F). Consistently, qPCR results showed that the mRNA expression of YAP downstream target genes cellular communication network factor 1 (Ccn1), cellular communication network factor 2 (Ccn2), and ankyrin repeat domain-containing protein 1 (Ankrd1) was significantly decreased in the EA group compared with the model group (*P* < 0.01), indicating that EA suppressed YAP transcriptional activity (Fig. 7G). To intuitively observe the effect of EA on YAP protein, immunofluorescence experiments showed that YAP protein in tumor tissues of the model group was mainly expressed in the nucleus, while YAP protein in the EA group was mainly expressed in the cytoplasm, indicating that EA intervention promoted the phosphorylation of YAP at the S127 site and inhibited its pro-cancer transcriptional activity (Fig. 7H).

### NGF promotes malignant behaviors of TNBC cells by inhibiting Hippo pathway

**Fig. 8:**
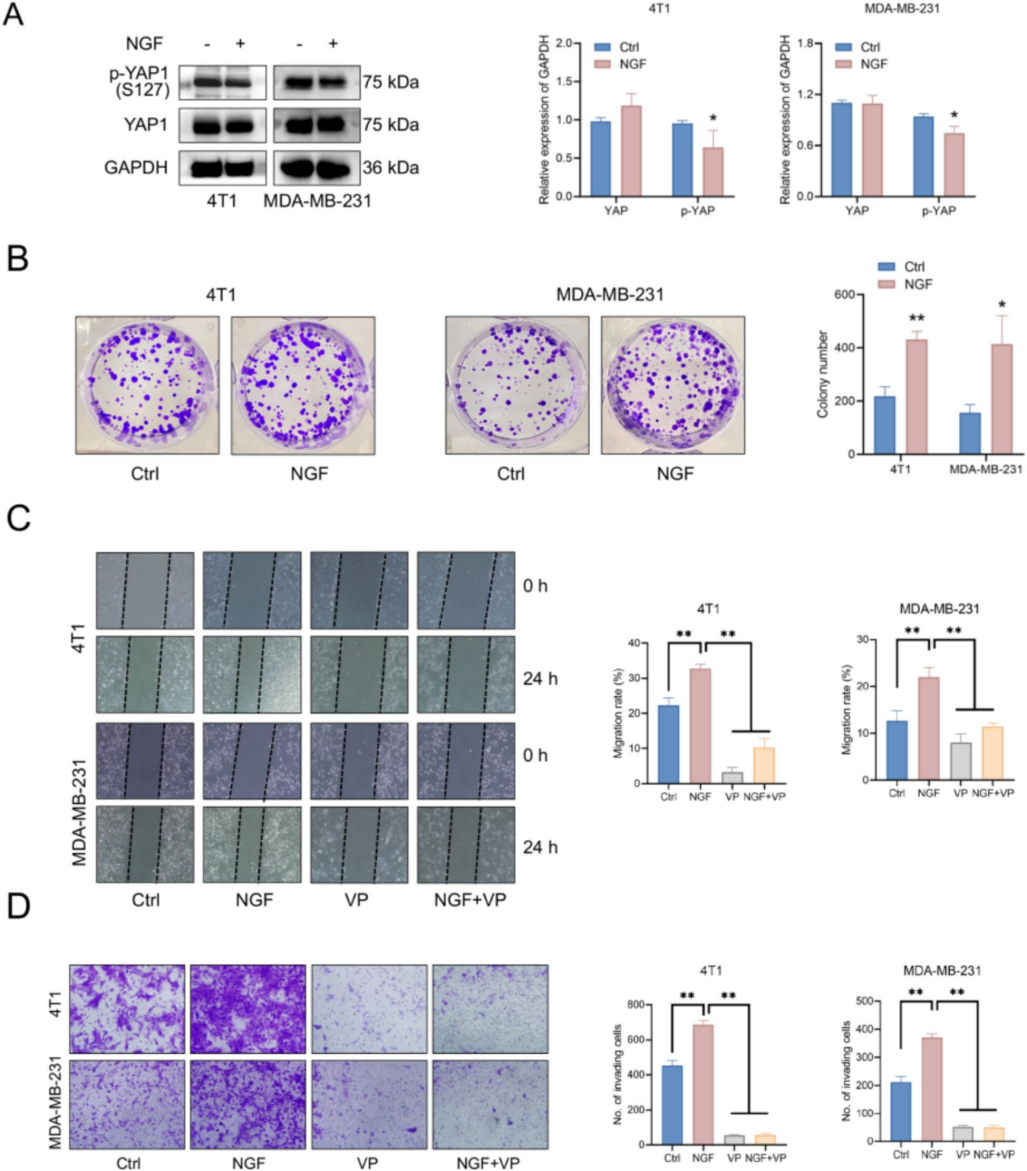
NGF inhibits the Hippo signaling pathway and promotes the malignant phenotype of TNBC. **A** Western blot detection of YAP1 protein expression and p-YAP1-S127 level in 4T1 and MDA-MB-231 cells. **B** Colony formation assay to detect the colony-forming ability of 4T1 and MDA-MB-231 cells. **C** Wound healing assay to detect the migration ability of 4T1 and MDA-MB-231 cells. **D** Transwell assay to detect the invasion ability of 4T1 and MDA-MB-231 cells. *P < 0.05, **P < 0.01, *n* = 3

Existing studies have shown that NGF can promote the invasion and migration of cervical cancer cells and the growth of xenograft tumors by inhibiting the Hippo pathway, and the YAP inhibitor verteporfin (VP) can eliminate the effect of NGF [40]. To explore the effect of NGF on the Hippo signaling pathway in TNBC, in vitro experiments were conducted. After treating 4T1 and MDA-MB-231 cells with β-NGF (200 ng/ml) for 24 h, the expression of total YAP1 protein showed no obvious change, but the phosphorylation level at its S127 site was significantly decreased (*P* < 0.05) (Fig. 8A). Cell colony formation experiments showed that after treating 4T1 and MDA-MB-231 cells with β-NGF for 24 h, the colony-forming ability of both 4T1 and MDA-MB-231 cells was significantly increased (*P* < 0.05; *P* < 0.01) (Fig. 8B). NGF intervention for 24 h could increase the migration and invasion abilities of 4T1 and MDA-MB-231 cells; however, after using VP (2 μm), the promoting effect of NGF on the malignant biological behavior of 4T1 and MDA-MB-231 cells was eliminated (*P* < 0.05; *P* < 0.01) (Figs. 8C–D).

### EA enhances T cell activation functions and inhibits IL-6 via NGF-Hippo axis in TNBC

**Fig. 9:**
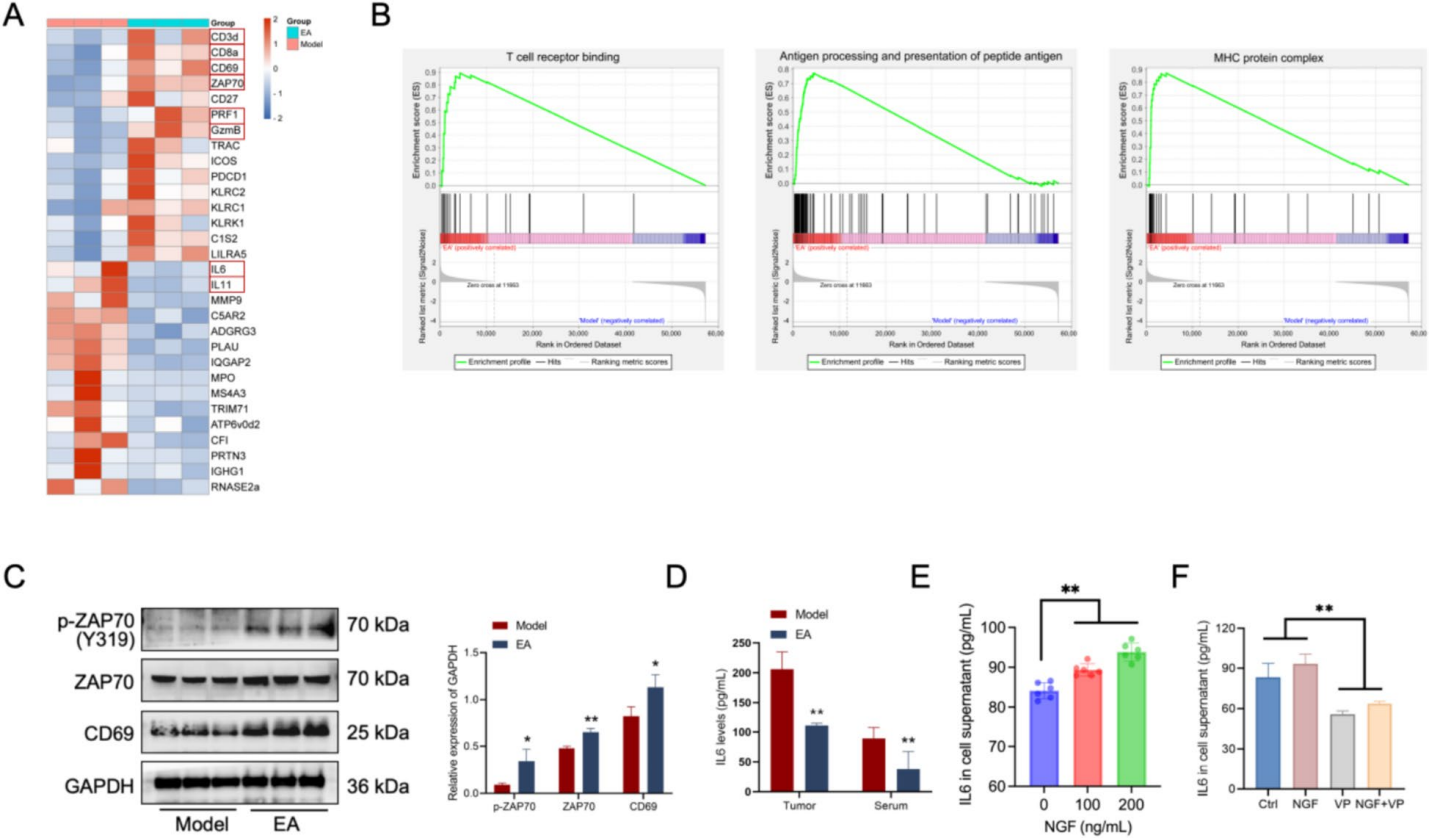
EA enhances T Cell function and inhibits IL-6 through NGF-Hippo axis in TNBC. **A** Heatmap showing the regulatory effect of EA on tumor immune-related genes in TNBC mice. **B** Gene Set Enrichment Analysis (GSEA) of the effect of EA on immune-related processes in the tumor immune microenvironment of TNBC mice. **C** Western blot detection of CD69, ZAP70 protein expression and ZAP70 phosphorylation level in tumor tissues, with three lanes per group representing individual biological replicates. **D** ELISA detection of IL-6 level in serum and tumor tissues. **E** The dose-dependent promotion of IL-6 secretion by NGF in 4T1 cells. **F** In vitro experiment to observe the inhibitory effect of YAP inhibitor verteporfin (VP) on NGF-induced promotion of IL-6 secretion. *P < 0.05, **P < 0.01, *n* = 3

The above studies found that EA could improve the tumor immune function of TNBC and increase the infiltration level of CD8^+^ T cells in tumor tissues. Therefore, based on the transcriptome sequencing results of tumor tissues, we analyzed its underlying mechanism at the gene level. The results showed that EA could directly increase the expression of CD3d (*FC* = 2.622, *P* = 0.037) and CD8α (*FC* = 2.847, *P* = 0.013) in tumor tissues of TNBC mice, upregulate the expression of T cell activation molecules CD69 (*FC* = 2.282, *P* = 0.019) and zeta chain of T cell receptor associated protein kinase 70 (ZAP70) (*FC* = 2.413, *P* = 0.035), and promote the secretion of T cell effector molecules PRF1 (*FC* = 3.168, *P* < 0.001) and GzmB (*FC* = 4.3205, *P* < 0.001). In addition, EA could downregulate the expression of immunosuppressive inflammatory factors IL-6 (*FC* = 0.342, *P* = 0.001) and IL-11 (*FC* = 0.418, *P* < 0.001) (Fig. 9A). Gene Set Enrichment Analysis (GSEA) showed that EA could upregulate immune processes such as the T cell receptor binding pathway (TCR) (*NES* = 1.857, *P* < 0.001), antigen processing and presentation of peptide antigen pathway (*NES* = 1.988, *P* < 0.001), and major histocompatibility complex (MHC) protein complex (*NES* = 1.992, *P* < 0.001) (Fig. 9B). Western blot experiments were performed to verify the transcriptome sequencing results, showing that EA could increase the protein expression of CD69 and enhance the protein expression of ZAP70 and its phosphorylation level (*P* < 0.05; *P* < 0.01) (Fig. 9C). ELISA results showed that the IL-6 level in the peripheral blood and tumor tissues of mice was significantly decreased after EA (*P* < 0.05) (Fig. 9D). Studies have found that in colorectal cancer, NE induces an increase in NGF, activating accelerated tumor growth [41]. It has also been reported in the literature that NE can activate IL-6, and the two form a positive feedback loop through gating reflex, jointly constituting a neuro-immune-endocrine regulatory axis [42]; in breast cancer, NE can promote IL-6 secretion through adrenoceptors (ARs) in breast cancer cells [43]. To determine whether NGF directly promotes IL-6 secretion in TNBC cells, 4T1 cells were treated with β-NGF at 0, 100, and 200 ng/mL for 24 h, and IL-6 levels in the culture supernatant were quantified by ELISA. The results demonstrated that NGF stimulated IL-6 secretion in a dose-dependent manner, with statistically significant differences observed between all three groups (*P* < 0.01), confirming that NGF promotes IL-6 secretion in TNBC cells in a concentration-dependent fashion (Fig. 9E). Further observation of whether NGF affects IL-6 secretion through the Hippo pathway found that when the YAP inhibitor VP was used, the promoting effect of NGF on IL-6 secretion disappeared (*P* < 0.01) (Fig. 9F). The above results indicate that NGF may affect IL-6 secretion through the Hippo pathway.

### α2-AR mediates NE-induced NGF/Hippo regulation in TNBC

**Fig. 10:**
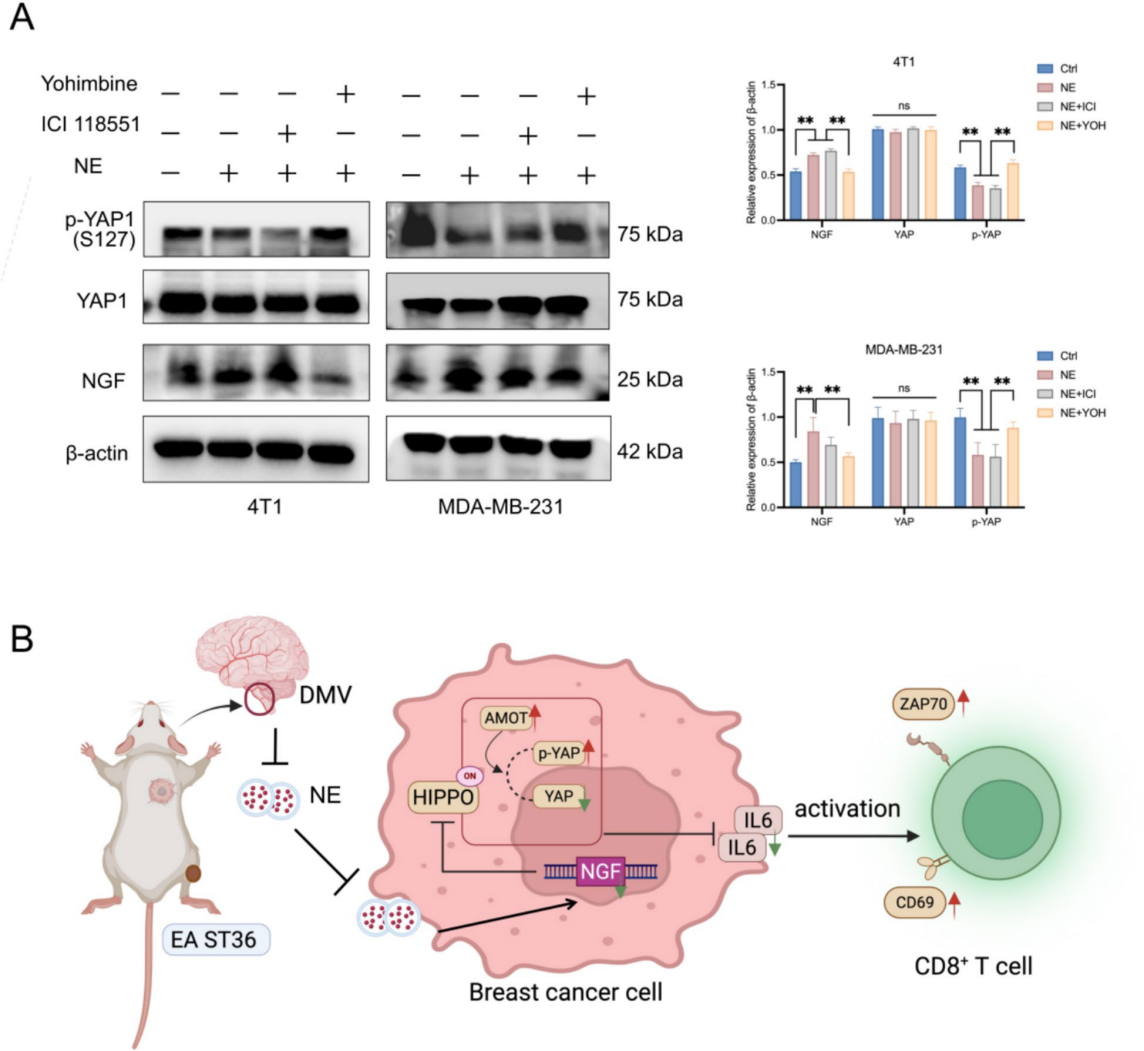
EA blocks α2-AR-mediated NE/NGF/Hippo axis to boost CD8^+^ T cell anti-tumor effect in TNBC. **A** Western blot detection of NGF, YAP protein expression and p-YAP1-S127 level. **B** Schematic diagram of the mechanism. *P < 0.05, **P < 0.01, *ns:* no significance, *n* = 3

To determine which adrenoceptor subtype is responsible for NE-mediated NGF upregulation, each adrenoceptor subtype was co-treated with its antagonist. NE (10 μM) was co-administered with the selective α2-AR antagonist Yohimbine (5 μM) or β2-AR antagonist ICI118551 (25 μM) to intervene in TNBC cells for 24 h. The NE-mediated increase in NGF protein expression in TNBC was rescued by Yohimbine (Fig. 10A). This indicates that the effect of NE on the NGF/Hippo signal is mediated by α2-AR. In summary, we found that EA at ST36 activates the vagus nerve, reduces NE release, blocks the α2-AR-mediated NE/NGF/Hippo pathway, thereby inhibiting immunosuppressive factors such as IL-6, and ultimately enhances the anti-tumor function of CD8^+^ T cells and potentiates with PD-L1 inhibitors (Fig. 10B).

## Discussion

Recognized in TCM as a crucial health-preserving acupoint [44, 45], ST36 has long been clinically acknowledged for its ability to tonify qi and blood, warm the middle energizer to dispel cold, and strengthen the body’s resistance against pathogens. Its therapeutic applications are extensive, covering digestive system diseases, postoperative rehabilitation, and immune regulation [46–48]. Preclinical research consistently highlights ST36’s multi-target potential in inhibiting malignant tumors and modulating tumor immunity, often involving multi-level regulation of the NEI network. For instance, in TNBC mouse models, EA at ST36 significantly enhances the proportion and cytotoxicity of CD8^+^ T cells and NK cells, concurrently reducing the accumulation and immunosuppressive activity of myeloid-derived suppressor cells (MDSCs). This tumor-inhibiting effect is notably abrogated by subdiaphragmatic vagotomy [11].

Song et al. [49, 50] investigated the effect of moxibustion at ST36 and BL18 (Ganshu) on a nude mouse model of colorectal cancer liver metastasis. They found that moxibustion significantly reduced the incidence of liver metastasis and the number of metastatic foci, with the potential mechanism linked to the regulation of intestinal microbiota imbalance. In lung cancer models, moxibustion at ST36 promotes DC activation and maturation in peritumoral lymph nodes, modulates CD4^+^, CD8^+^ T cell, and NK cell levels, and inhibits tumor growth in an NK cell-dependent fashion [51]. Additionally, acupuncture involving ST36, GV14 (Dazhui), BL17 (Geshu), and BL23 (Shenshu) reduces the co-expression of PD-1 and T cell immunoglobulin and mucin domain-containing protein 3 (TIM3) on tumor-infiltrating CD8^+^ T cells, thereby ameliorating the tumor immune microenvironment in melanoma and colorectal cancer mouse models [14]. Furthermore, EA at ST36 has been shown to inhibit tumor burden in inflammation-associated colorectal cancer mouse models, decreasing serum levels of IL-6, tumor necrosis factor-alpha (TNF-α), IL-17A, and IL-23, and delaying colonic inflammation-cancer transformation [52]. The therapeutic efficacy of EA is known to be influenced by intervention intensity and timing [53]. For instance, Wan et al. [54] observed that EA initiated on day 8 post-modeling yielded optimal tumor growth inhibition in breast cancer-bearing mice, correlating with an increased proportion of peripheral blood CD8^+^ T cells and NK cells. Similarly, varying EA parameters differentially impacted Lewis lung cancer subcutaneous xenograft growth, with 10 Hz frequency and 2 mA intensity demonstrating the most significant tumor-inhibiting effect [55]. Drawing upon previous research, our study established a systematic parameter-screened EA protocol (2/15 Hz, 3 mA, 30 min). Although EA intervention did not significantly reduce spleen size or weight compared to the model group, this finding is not indicative of immunological inefficacy. This is consistent with EA's predominant immunological activity being localized to the tumor microenvironment, as demonstrated by the intratumoral enrichment of CD8^+^ T cells, NK cells, DCs, and M1 macrophages. We validated that ST36 stimulation inhibits TNBC tumor progression and exhibits a synergistic anti-tumor effect when combined with αPD-L1, crucially dependent on CD8^+^ T cell function. This highlights EA’s promising potential as an adjuvant therapy with immune checkpoint inhibitors.

At the neuroregulation level, our detection found that EA at ST36 could activate c-Fos^+^/ChAT^+^ neurons in the DMV region and drive vagal nerve conduction. As the main neurotransmitter of the vagus nerve, the expression of Ach in peripheral blood increased after EA, while the Ach content in tumor tissues decreased. The sympathetic marker NE was also decreased by EA in both peripheral blood and tumor tissues. Ach can synergistically promote the progression of malignant tumors through multiple pathways. In a mouse breast cancer model, Ach remodels the pre-metastatic niche of breast cancer by enhancing NETosis, promoting breast cancer lung metastasis [56]. In pancreatic ductal adenocarcinoma, ACh directly inhibits IFN-γ production by CD8^+^ T cells in a dose-dependent manner; excessive activation of cholinergic signals promotes tumor growth by inhibiting intra-tumoral T cell responses [57]. The pro-proliferative effect of ACh on malignant tumors is related to the activation of the NGF/Hippo signaling pathway. Ach stimulation induces NGF expression in gastric epithelium, promotes abnormal proliferation of intestinal nerves, and accelerates the carcinogenesis process. Blocking NGF and downstream signals inhibits epithelial proliferation and tumorigenesis in a muscarinic acetylcholine receptor 3 (M3R)-dependent manner, partly by inhibiting YAP function [58]. NE also plays a key regulatory role in the occurrence and development of malignant tumors [59, 60]. NE induces the expression of Twist1, a key regulator of mesenchymal-like phenotype and epithelial-mesenchymal transition, in a β2-AR-dependent manner, promoting the migration of glioma cells [61]. Lung cancer patients with anti-PD-1 resistance have high plasma NE levels; in-depth exploration found that NE can affect the secretion of C-X-C motif chemokine ligand 9 (CXCL9) and adenosine (ADO) in tumor cells, thereby inhibiting the chemotaxis and function of CD8^+^ T cells and inducing anti-PD-1 resistance [62]. In this study, NE was selected as the main research direction; however, the “antagonistic-synergistic” relationship between NE and the vagus nerve/Ach in TNBC [58, 63, 64] still needs further research to clarify.

At the immune regulation level, transcriptome analysis confirmed the positive effect of EA on CD8^+^ T cell function and its effector factors PRF1 and GzmB, which was consistent with the experimental results. It was also found that EA was related to the upregulated expression of T cell activation molecules CD69 and ZAP70, and downregulated expression of immunosuppressive inflammatory factors IL-6 and IL-11. As an early marker of T cell activation, CD69 is rapidly expressed after CD8^+^ T cells recognize tumor antigens, and directly enhances the anti-tumor effect of CD8^+^ T cells by strengthening the TCR and promoting lymphocyte retention in tumor tissues [65, 66]. ZAP70 is a key kinase in the TCR signaling pathway; in CD8^+^ T cells, it initiates the activation cascade by phosphorylating downstream adapter proteins, which is crucial for the response to tumor antigens [67]. Defects in ZAP70 function will prevent CD8^+^ T cells from effectively recognizing and killing tumor cells, and its expression level is positively correlated with the responsiveness to immune checkpoint inhibitor treatment [68]. IL-6 is a classic immunosuppressive factor that can induce T cell dysfunction and promote the differentiation of regulatory T cells (Tregs), ultimately inhibiting anti-tumor immunity and the efficacy of immune checkpoint inhibitors [69, 70]. IL-11 can indirectly inhibit the infiltration and function of CD8^+^ T cells by inducing the expansion of MDSCs and polarization of M2-type macrophages, and can upregulate PD-L1 to promote immune escape [71]. In summary, EA reshapes the balance of anti-tumor immune response through the dual mechanisms of “enhancing effector cell activity” and “alleviating microenvironment inhibition.” This synergistic regulatory model not only provides new evidence for understanding the immunological basis of acupuncture anti-tumor, but also may provide new ideas for breaking through the current bottleneck of immunotherapy.

At the molecular mechanism level, we focused on the role of NE in TNBC tumor immunity and malignant phenotype through the NGF/Hippo pathway, and in-depth explored the mediating role of ARs. In in vitro experiments, NGF intervention increased the invasion, migration, and colony-forming abilities of TNBC cells, while this effect was blocked by the YAP inhibitor VP; meanwhile, the secretion of the immunosuppressive factor IL-6 also decreased. The relationship between NGF, the Hippo pathway, and YAP protein has also been reported in previous studies [40]. NGF inhibits the phosphorylation activity of the Hippo pathway kinase Large Tumor Suppressor Kinase 1 (LATS1) by activating its receptor Neurotrophic Receptor Tyrosine Kinase 1 (NTRK1), thereby reducing the phosphorylation of YAP at the S127 site, promoting YAP nuclear translocation and transcriptional activity, and ultimately driving tumor proliferation and migration [72].

As G protein-coupled receptors, ARs are a class of membrane protein receptors that mediate the physiological effects of catecholamines and play key regulatory roles in the cardiovascular, metabolic, and immune systems [73]. In mammals, there are 9 subtypes of ARs, including α1 (α1A, α1B, α1D), α2 (α2A, α2B, α2C), and β (β1, β2, β3) receptors. Each subtype exerts unique functions due to differences in tissue distribution and signaling pathways. α1 receptors are mainly distributed in vascular smooth muscle and pupillary dilator muscle, activating phospholipase C through guanine nucleotide regulatory protein (Gq) to mediate vasoconstriction and pupillary dilation; α2 receptors are mostly located on presynaptic membranes and adipocytes, inhibiting adenylate cyclase via inhibitory G protein (Gi) to regulate neurotransmitter release and lipid metabolism; β receptors are widely distributed in cardiac muscle, bronchi, and adipose tissue–β1 receptors enhance myocardial contractility through stimulatory adenylate cyclase-activating G protein (Gs), β2 receptors mediate bronchial dilation and vascular smooth muscle relaxation, and β3 receptors are specifically involved in lipolysis and thermogenesis. Based on current studies, β2-AR and α2-AR are the two most significant subtypes with the clearest mechanisms in the regulation of tumor immunity by NE, and they exert opposite immune regulatory functions in the tumor microenvironment. In colorectal cancer, NE can activate the downstream protein kinase A (PKA)–cAMP response element-binding protein (CREB) and mitogen-activated protein kinase (MAPK) pathways through β2-AR, increasing NGF secretion to promote sympathetic innervation in tumors; NGF further promotes the accumulation of NE, and the two form a positive feedback loop. NE and NGF promote and accelerate tumor progression through the α2-AR/Gi protein-YAP signaling pathway and Tropomyosin Receptor Kinase A (TrkA)-phosphatidylinositol 3-kinase (PI3K)/Protein Kinase B (AKT) signaling pathway, respectively. Consistent with this, in our TNBC cell model studies, we observed that NE could significantly inhibit the phosphorylation of YAP, and this effect could be reversed by α2-AR-specific inhibitors. However, in vitro studies lack the dynamic regulation of nerve fiber innervation in the in vivo nerve-tumor microenvironment and its interaction with other neurotransmitters and immune cell infiltration; the in-depth mechanism needs further exploration.

A notable limitation of the present study is the exclusive use of the 4T1 cell line for TNBC modeling, which restricts the generalizability of our findings to a single syngeneic model. To address this gap, future studies will supplement experiments with additional TNBC cell lines as well as patient-derived xenograft (PDX) models. On the other hand, although NE was the neurotransmitter we focused on in-depth in this study, the differential changes in ACh levels across various tissues following EA treatment represent a highly intriguing phenomenon. Tumor cells, various immune cells within the TME and normal tissues exhibit distinct patterns and levels of muscarinic and nicotinic acetylcholine receptors. EA might selectively modulate ACh release or its downstream effects based on these tissue-specific receptor profiles. Future studies are crucial to elucidate these mechanisms, including detailed exploration of the expression and functional activity of specific muscarinic and nicotinic ACh receptors in TNBC tissues, as well as the impact of EA on key enzymes involved in ACh synthesis and degradation within the tumor microenvironment.

In summary, this study revealed that EA activates c-Fos^+^/ChAT^+^ neurons in the DMV region, reduces NE secretion, and effectively blocks the immunosuppressive effect of the NE/α2-AR/NGF/Hippo pathway. These findings not only challenge the traditional understanding that acupuncture merely alleviates symptoms but also unequivocally clarify its direct intervention in the tumor process through neuro-endocrine-immune three-level regulation. Ultimately, this work provides a molecular biological basis for the anti-tumor effects of acupuncture, supporting its potential as an adjuvant tumor therapeutic strategy.

## Supplementary Information

Additional file 1 (XLSX 52 KB)

Additional file 2 (XLSX 10 KB)

## Data Availability

No datasets were generated or analysed during the current study.

## Abbreviations

Ach: Acetylcholine
AMOT: Angiomotin
AR: Adrenergic Receptor
α2-AR: ‌α2-Adrenergic Receptor
β2-AR: β2-Adrenergic Receptor
BCA: Bicinchoninic Acida
ChAT: Choline Acetyltransferase
CMAP: Compound Muscle Action Potential
Ctrl: Control
DC: Dendritic Cell
DEG: Differentially Expressed Gene
DMV: Dorsal Motor Nucleus of the Vagus nerve
DTR: Deep Tendon Reflex
EA: Electroacupuncture
EPI/Epi: Epinephrine
ELISA: Enzyme-Linked Immunosorbent Assay
FC: Fold Change
FBS: Fetal Bovine Serum
GEPIA: Gene Expression Profiling Interactive Analysis
GSEA: Gene Set Enrichment Analysis
GO: Gene Ontology
HR: Hazard Ratio
ICIs: Immune Checkpoint Inhibitors
IL: Interleukin
KEGG: Kyoto Encyclopedia of Genes and Genomes
LATS1: Large Tumor Suppressor Kinase 1
LLN: Lower Limit of Normal
M1: M1-type macrophage
M2: M2-type macrophage
MRC: Medical Research Council
MDSC: Myeloid-Derived Suppressor Cell
mRNA: Messenger Ribonucleic Acid
NE: Norepinephrine
NGF: Nerve Growth Factor
NTRK1: Neurotrophic Receptor Tyrosine Kinase 1
ns: not significant
PD-1: Programmed Death-1
PD-L1: Programmed Death Ligand 1
αPD-L1: anti-PD-L1 antibody
PRF: Perforin
PMSF: Phenylmethylsulfonyl Fluoride
PVDF: Polyvinylidene Fluoride
p-YAP1-S127: Ser127-phosphorylated YAP1
qPCR: Quantitative Real-time PCR
RFS: Recurrence-Free Survival
RIPA: Radio-Immunoprecipitation Assay
SAP: Sensory Action Potential
SRA: Sequence Read Archive
ST25: Tianshu acupoint
ST36: Zusanli acupoint
TCM: Traditional Chinese Medicine
TCR: T Cell Receptor
TIL: Tumor-Infiltrating Lymphocyte
TME: Tumor Microenvironment
TNBC: Triple-Negative Breast Cancer
TUNEL: Terminal deoxynucleotidyl transferase dUTP nick end labeling
ULN: Upper Limit of Normal
VP: Verteporfin
WB: Western blot
YAP: Yes-Associated Protein
ZAP70: Zeta chain of T cell receptor associated protein kinase 70
GzmB: Granzyme B
